# Genome-wide CRISPR screening identifies a role for ARRDC3 in TRP53-mediated responses

**DOI:** 10.1038/s41418-023-01249-3

**Published:** 2023-12-14

**Authors:** John E. La Marca, Brandon J. Aubrey, Bruce Yang, Catherine Chang, Zilu Wang, Andrew Kueh, Lin Tai, Stephen Wilcox, Liz Milla, Susanne Heinzel, David Vremec, Lauren Whelan, Christina König, Deeksha Kaloni, Anne K. Voss, Andreas Strasser, Sarah T. Diepstraten, Marco J. Herold, Gemma L. Kelly

**Affiliations:** 1 The Walter and Eliza Hall Institute, Parkville, Victoria Australia; 2 Department of Medical Biology, University of Melbourne, Parkville, Victoria Australia; 3 Genome Engineering and Cancer Modelling Program, Olivia Newton-John Cancer Research Institute, Melbourne, Victoria Australia; 4 School of Cancer Medicine, La Trobe University, Melbourne, Victoria Australia; 5 Department of Medicine, Massachusetts General Hospital, Boston, USA; 6 Environomics Future Science Platform, Centre for Australian National Biodiversity Research, CSIRO, Canberra, Australia

**Keywords:** Cancer, Cell biology

## Abstract

Whole-genome screens using CRISPR technologies are powerful tools to identify novel tumour suppressors as well as factors that impact responses of malignant cells to anti-cancer agents. Applying this methodology to lymphoma cells, we conducted a genome-wide screen to identify novel inhibitors of tumour expansion that are induced by the tumour suppressor TRP53. We discovered that the absence of Arrestin domain containing 3 (ARRDC3) increases the survival and long-term competitiveness of MYC-driven lymphoma cells when treated with anti-cancer agents that activate TRP53. Deleting *Arrdc3* in mice caused perinatal lethality due to various developmental abnormalities, including cardiac defects. Notably, the absence of ARRDC3 markedly accelerated MYC-driven lymphoma development. Thus, ARRDC3 is a new mediator of TRP53-mediated suppression of tumour expansion, and this discovery may open new avenues to harness this process for cancer therapy.

## Introduction

The intrinsic apoptosis pathway is a tightly regulated process necessary for normal development and tissue homeostasis, but can also be activated by external cellular stress, such as radiation or nutrient deprivation [1]. The first step in intrinsic apoptosis signalling is the transcriptional and/or post-transcriptional upregulation of pro-apoptotic BH3-only proteins (e.g. PUMA, NOXA, BIM) in response to, for example, activation of the tumour suppressor TRP53 [2–5]. BH3-only proteins can bind and inhibit the pro-survival BCL-2 proteins (e.g. BCL-2, BCL-XL, MCL-1), resulting in the release and activation of the pro-apoptotic effectors BAK and BAX, though some BH3-only proteins have been reported to also activate BAK/BAX directly [6]. Activated BAK/BAX oligomerise and form pores in the mitochondrial outer membrane (MOMP), allowing release of apoptogenic factors from the space between the inner and outer mitochondrial membranes. This instigates apoptosome formation and activation of the caspase cascade, effecting cell destruction.

Mutated in more than 50% of all human cancers, the transcription factor TP53 (called TRP53 in mice) is a critical tumour suppressor [7]. Moreover, the efficacy of many chemotherapeutic drugs, particularly those that cause DNA damage (e.g., etoposide, cisplatin), depends upon intact TP53/TRP53 functionality, consequently meaning that mutant TP53 cancers often respond poorly to cancer therapy [8]. In stressed cells, TP53/TRP53 controls the expression of genes that regulate cellular responses that cooperate to suppress tumour development, such as apoptotic cell death, cell cycle arrest and senescence, coordination of DNA repair, and adaptation of cellular metabolism [7]. Several TP53/TRP53 target genes critical for some of these processes have been identified, such as those encoding the pro-apoptotic BH3-only proteins PUMA and NOXA (*BBC3* and *PMAIP1*, respectively) [2, 3, 5], as well as the cyclin-dependent kinase inhibitor P21/*CDKN1A* [9]. Notably, however, mice deficient for all three of these genes—*Bbc3*, *Pmaip1*, and *Cdkn1a*—do not spontaneously develop tumours [10], in striking contrast to the highly tumour prone *Trp53* knockout mice [11]. This indicates that additional target genes, and the processes they operate in, must play critical roles in TP53/TRP53-mediated tumour suppression. For example, in vivo shRNA screens have identified DNA repair genes (e.g. *MLH1*) as important effectors of TP53/TRP53-mediated tumour suppression [12].

In this study, we aimed to identify novel negative regulators of lymphoma cell expansion/survival that function downstream of TP53/TRP53 activation. Such regulators could be potential therapeutic targets that, when activated, bypass drug resistance caused by mutation or loss of TP53/TRP53. To this end, we conducted CRISPR/Cas9 whole-genome screens to identify novel genes that mediate TRP53-tumour suppressive responses in murine *Eμ-Myc* lymphoma cells, a well-established model of aggressive B cell lymphoma [13]. We identified and validated *arrestin domain containing 3* (*Arrdc3*), the loss of which provided a competitive growth/survival advantage to lymphoma cells after TRP53 activation. ARRDC3 (also known as TBP-2-like inducible membrane protein (TLIMP)) is one of six α-arrestins, with 2 visual- and 2 β-arrestins rounding out the wider arrestin family [14]. ARRDC3 has most commonly been identified in roles also ascribed to other, better-studied arrestins, such as in the suppression of G-protein coupled receptor (GPCR) signalling, where it mediates receptor ubiquitination and lysosomal degradation [15–21]. Several publications have associated abnormalities in ARRDC3 with a wide variety of cancers, particularly in epithelial to mesenchymal transition (EMT) and invasiveness, where loss/downregulation of ARRDC3 contributes to more severe phenotypes [22–28]. However, to our knowledge, ARRDC3 has not previously been described in the context of TP53-mediated tumour suppression. Extending our discovery, we generated *Arrdc3* knockout mice, which revealed that *Arrdc3* is an essential gene, as *Arrdc3*^−*/*−^ mice died perinatally. Interestingly, transplanting lethally irradiated mice with foetal liver cells from E14.5 *Eμ-Myc*^*T/+*^*;Arrdc3*^*−/−*^ or control *Eµ-Myc*^*T/+*^ foetuses demonstrated that loss of ARRDC3 greatly accelerated MYC-driven lymphoma development. Altogether, our research demonstrates that *Arrdc3* is an essential gene, and plays an important role in TRP53-mediated suppression of MYC-driven lymphoma development.

## Results

### CRISPR/Cas9 screening indicates loss of ARRDC3 provides a competitive advantage to *Eμ-Myc* lymphoma cells after TRP53 activation

**Fig. 1:**
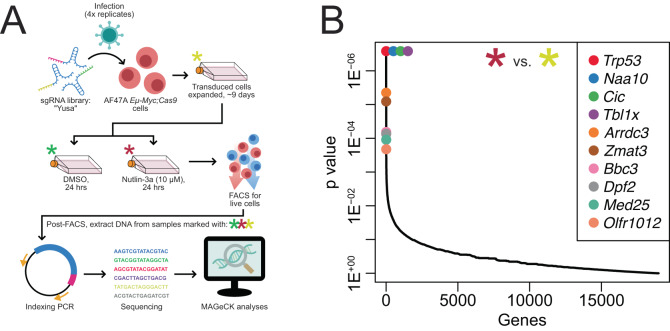
*Arrdc3* identified as a hit in a CRISPR/Cas9 screen for regulators of TRP53-mediated lymphoma growth suppression. **A** Diagram of the CRISPR screen methodology using the MDM2 inhibitor nutlin-3a, which activates TP53/TRP53 in a non-genotoxic manner, as a selection pressure. In quadruplicate, AF47A *Eμ-Myc* lymphoma cells were transduced with the whole-genome “Yusa” sgRNA library, expanded, and either cultured without treatment or treated with either DMSO (vehicle control) or nutlin-3a (10 μM) for 24 h. Live cells were then sorted by FACS, DNA extracted from each sample (marked by asterisks), indexing PCR performed, and NGS combined with bioinformatic analyses used to identify sgRNAs that conferred a survival/competitive advantage after TRP53 activation. **B** Top hits from the CRISPR screen, comparing the untreated control cells (yellow asterisk) with the cells treated with nutlin-3a (red asterisk). *Arrdc3* was identified as one of the top hits, with *Arrdc3* targeting sgRNAs highly enriched in the surviving nutlin-3a-treated lymphoma cells.

Using *Eμ-Myc* lymphoma-derived cell lines, we performed a CRISPR/Cas9 screen with the mouse whole-genome “Yusa” sgRNA library [29], and utilised the MDM2 inhibitor nutlin-3a to activate TRP53 in a non-genotoxic manner. Specifically, *Eμ-Myc* lymphoma cells stably transduced with a Cas9 expression vector were further transduced with the “Yusa” sgRNA library and expanded for nine days before being separated into two streams—24 h treatment with DMSO (vehicle control) or nutlin-3a (Fig. 1A). Surviving cells were sorted by FACS, their genomic DNA extracted, and next generation sequencing (NGS) undertaken. Bioinformatic analyses were then performed to identify the enriched sgRNAs in each of the streams of our experiment. When comparing the nutlin-3a-treated cells to the untreated control cells, loss of *Trp53* was, as expected, the top hit (Fig. 1B). This, along with loss of the apoptosis mediator *Bbc3*/PUMA also being an expected strong hit [30], provided strong validation of our screening approach. This same comparison also returned *Arrdc3* as the 5th top hit (Fig. 1B), suggesting that loss of *Arrdc3* confers a survival/growth advantage in *Eμ-Myc* lymphoma cells treated with the TP53/TRP53-activating drug nutlin-3a. Interestingly, when comparing the nutlin-3a-treated samples to the DMSO-treated samples, *Arrdc3* dropped to the 21^st^ top hit. This indicates that there was also some low-level selection for loss of *Arrdc3* in the DMSO-treated samples (which had also undergone the additional process of cell sorting) compared to the untreated samples. This likely indicates that *Arrdc3* loss may generally enhance the survival/growth of *Eµ-Myc* lymphoma cells, which are highly apoptosis-prone, under normal (or slightly stressful; e.g. DMSO treatment/cell sorting) conditions (Figures S1A, B). Since *Arrdc3* was more strongly enriched in the nutlin-3a-treated samples than the DMSO-treated samples, we chose to pursue the validation of *Arrdc3* as a potential factor in TRP53-mediated tumour growth suppressing responses.

### Validation that *Arrdc3* is a TRP53-target gene, and loss of *ARRDC3* provides a competitive advantage to *Eμ-Myc* lymphoma cells treated with TRP53-activating drugs

To validate *Arrdc3* as a hit from our screen, we used CRISPR/Cas9 and an sgRNA targeting *Arrdc3* to generate *Arrdc3* knockout (*Arrdc3*^*KO*^) cells in three well-characterised *Eμ-Myc* mouse lymphoma cell lines—AH15A, AF47A, and 560 [31]. The efficacy of *Arrdc3* disruption was confirmed by NGS (Figure S2).

**Fig. 2:**
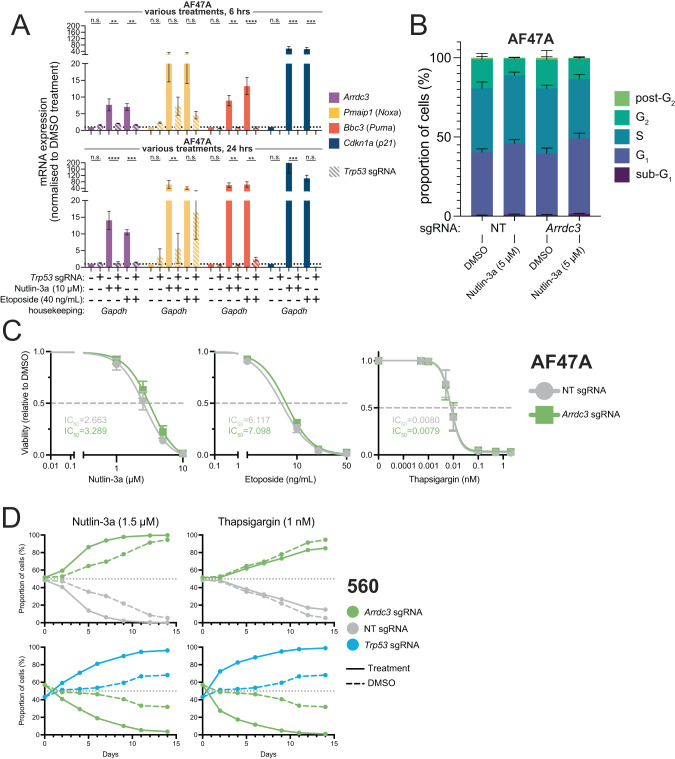
*Arrdc3* was validated as a TRP53 regulated gene and loss of *Arrdc3* provided a competitive advantage to *Eμ-Myc* lymphoma cells after TRP53 activation. **A** qRT-PCR analysis to assess the levels of *Arrdc3* expression in AF47A *Eμ-Myc* lymphoma cells after treatment for 6 or 24 h with nutlin-3a (10 μM) or etoposide (40 ng/mL), relative to expression in DMSO-treated control cells. Additionally, expression of *Pmaip1*, *Bbc3*, and *Cdkn1a* were assessed as controls, and the expression of each gene was also examined in *Trp53*^*KO*^ cells to assess the level of reliance of *Arrdc3* expression on TRP53 activity. *Gapdh* was used as a housekeeping gene. Each treatment was performed three times, and the qRT-PCR was undertaken with 3 technical replicates. Error bars represent standard error of the mean. Statistical tests were one-way ANOVAs with Šídák’s multiple comparisons tests, performed to compare the indicated samples. Note that due to variation between biological replicates, some differences are not statistically significant despite obvious trends. **B** Cell cycle assay using the AF47A *Eμ-Myc* lymphoma cell line with CRISPR/Cas9-mediated knockout of *Arrdc3* compared to the NTsgRNA transduced control lymphoma line, treated with 5 μM nutlin-3a for 6 h. Each treatment was performed 3 times. Data are presented as means +/− standard deviation. Statistical analyses are given in Table S1. **C** Cell death assays using the AF47A *Eμ-Myc* lymphoma cell line edited using CRISPR/Cas9 to ablate *Arrdc3*, treated for 24 h with one of three apoptosis-inducing drugs: nutlin-3a and etoposide that act via TP53/TRP53, or thapsigargin that functions in a TP53/TRP53-independent manner. Each cell line treatment was performed 3 times, with 2 technical replicates each time. Data are presented as means +/− standard deviation. **D** Cell competition assays using the 560 *Eμ-Myc* lymphoma cell line with CRISPR/Cas9-mediated knockout derivative lines of either *Arrdc3* or *Trp53*, or a non-targeting sgRNA (NTsgRNA). Mixed cell populations (1:1) were treated with sub-optimal doses of either nutlin-3a (1.5 µM, ~IC_20_) or thapsigargin (1 nM, ~IC_90_) over a period of 14 days. Each competition assay was performed twice for each cell line, with 2 technical replicates each time, with a representative example being shown. Data are presented as means +/− standard deviation.

We first hypothesised that *Arrdc3* might be a transcriptional target (direct or indirect) of the master regulator TRP53. As such, we examined the expression of *Arrdc3*, and as positive controls the well-known TRP53 targets *Pmaip1* (encodes NOXA), *Bbc3* (encodes PUMA), and *Cdkn1a* (encodes p21), in isogenic AF47A *Eμ-Myc* lymphoma cells with a non-targeting sgRNA (NTsgRNA) [32] or made *Trp53*^*KO*^ by CRISPR/Cas9 (previously validated [31]) after 6 and 24 h of treatment with nutlin-3a or etoposide (Figs. 2A, S3). While baseline *Arrdc3* expression levels were similar between untreated control and *Trp53*^*KO*^ *Eμ-Myc* lymphoma cells, there was a marked increase in *Arrdc3* expression after treatment with nutlin-3a in the NTsgRNA *Eμ-Myc* lymphoma cells (~7–17-fold induction over 6–24 h) and treatment with etoposide (~5–10-fold induction over 6–24 h), but no such increase was seen in the *Trp53*^*KO*^ *Eμ-Myc* lymphoma cells (Figs. 2A, S3). As expected, we observed marked increases in the expression of the positive control TRP53 target genes in the NTsgRNA cells, but not in the *Trp53*^*KO*^ cells, after both treatments. These data demonstrate that *Arrdc3* can be transcriptionally regulated by TRP53 in *Eμ-Myc* lymphoma cells. Whether this transcriptional regulation is direct or indirect remains unclear.

We next examined whether *Arrdc3* loss affected the proliferation rate/cycling of *Eμ-Myc* lymphoma cells after TRP53 activation. We treated the isogenic NTsgRNA control and *Arrdc3*^*KO*^ *Eμ-Myc* lymphoma cell lines with 5 μM nutlin-3a (~IC_85_) for 6 h and assessed cell cycle stages by staining the DNA with DAPI. The observed reductions in the numbers of cells in S-phase and increases in the numbers of cells in the G_1_-phase, comparing nutlin-3a treated cells with DMSO (vehicle control) treated cells (Fig. 2B), is expected after TRP53 activation [13, 30, 33]. However, we could discern no consistent differences between the control cells or the *Arrdc3*^*KO*^ cells in all cell backgrounds (Figs. 2B, S4A; statistical analyses in Table S1). This demonstrates that ARRDC3 does not play a major role in TRP53-mediated cell cycle arrest.

Next, viability assays were undertaken on these *Arrdc3*^*KO*^ and NTsgRNA *Eμ-Myc* lymphoma cell lines. The lymphoma cells were treated for 24 h with increasing concentrations of nutlin-3a, etoposide (a DNA damaging agent that causes activation of TP53/TRP53), and thapsigargin (induces apoptosis in a TP53/TRP53-independent manner by causing endoplasmic reticulum stress [34]) (Figs. 2C, S4B, C). For both nutlin-3a and etoposide, we observed a slight but not statistically significant increase in the viability of *Arrdc3*^*KO*^ lymphoma cells compared to the NTsgRNA control lymphoma cells, while thapsigargin killed lymphoma cell lines of all genotypes to a similar extent. These data suggest that while *Arrdc3* might have a small role in TRP53-mediated apoptosis, it is likely not its predominant function. As a positive control, we also validated our hit of *Bbc3* (encoding PUMA) via 24 h viability assay, and observed resistance to nutlin-3a- and etoposide-mediated killing (Fig. S4D).

Finally, to mimic an in vivo scenario more closely, where only some cells possess a particular mutation, we examined whether *Arrdc3*^*KO*^ *Eμ-Myc* lymphoma cells had a competitive advantage over control *Eμ-Myc* lymphoma cells when grown in sub-lethal doses of these drugs over a longer period. To this end, we set our *Arrdc3*^*KO*^ *Eμ-Myc* lymphoma cells against their concomitant NTsgRNA *Eµ-Myc* controls in competition assays, and also in parallel against isogenic *Trp53*^*KO*^ *Eμ-Myc* lymphoma cells. These mixed lymphoma cell populations were then treated with either nutlin-3a (1.5 µM) or thapsigargin (1 nM), at doses chosen to kill significant proportions of the cells. In the *Arrdc3*^*KO*^ *vs* control lymphoma cell competition, we observed outgrowth of the *Arrdc3*^*KO*^ population over control cells even without any treatment, and this competitive advantage was enhanced in the presence of nutlin-3a (Figs. 2D, S4E). Interestingly, when treated with thapsigargin, *Arrdc3*^*KO*^ lymphoma cells did not exhibit a competitive advantage *vs* control lymphoma cells beyond that observed after DMSO treatment. This suggests that while ARRDC3 likely plays a role in TRP53-mediated suppression of lymphoma cell expansion, it may have only limited involvement in TRP53-independent suppression of lymphoma cell growth or survival. By contrast, *Trp53*^*KO*^ lymphoma cells outcompeted control and *Arrdc3*^*KO*^ lymphoma cells when treated with either nutlin-3a or thapsigargin in both the AH15A and 560 cell lines, though the *Arrdc3*^*KO*^ AF47A cells proved slightly more resilient (Figs. 2D, S4E). Overall, these data indicate that *Arrdc3* is a TRP53 target, and its loss gives cells a competitive advantage in the face of TRP53 activation, possibly via low-level apoptosis protection that can be selected for over time.

### *Arrdc3* is essential for normal mouse development

**Fig. 3:**
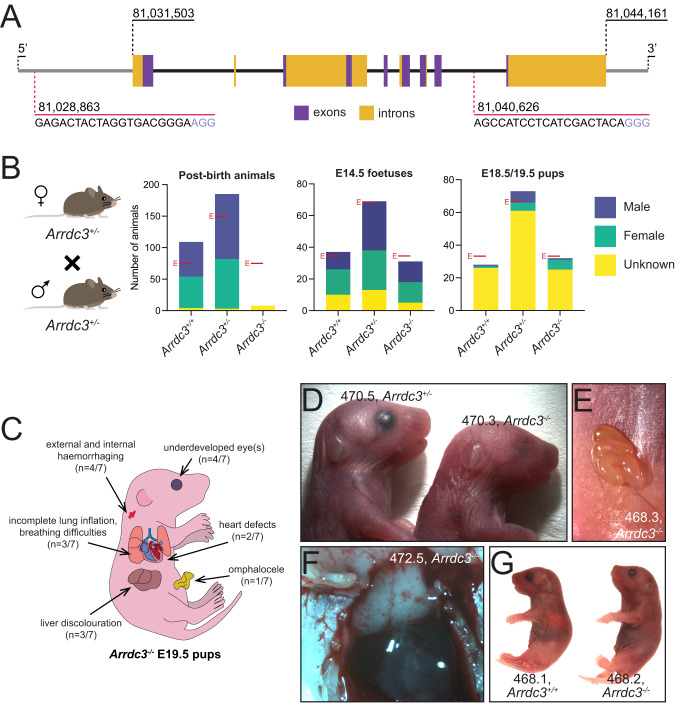
*Arrdc3* knockout mice are not viable, with a range of abnormalities evident at embryonic day E19.5. **A** The coding region of the *Arrdc3* gene spans the region 81,031,503…81,044,161 on chromosome 13 in mice. *Arrdc3* knockout mice were generated using CRISPR/Cas9 gene editing, with sgRNAs (red) targeting the 3’ UTR upstream of the gene and between exons (purple) 7 and 8, resulting in the deletion of the majority of the *Arrdc3* gene locus. **B** The genotypes of offspring of inter-crosses between *Arrdc3*^*+/−*^ mice do not obey Mendelian ratios post-birth (X^2^ = 82.87, df = 2, *p* < 0.05 (*p* < 1 × 10^−15^)), with only 8 *Arrdc3*^*−/−*^ offspring being observed among 302 animals, none of which survived past weaning. By contrast, E14.5 foetuses generated from inter-crosses of *Arrdc3*^*+/−*^ mice were seen to obey Mendelian ratios (X^2^ = 0.53, df = 2, *p* > 0.05 (*p* = 0.766)), with 31 of 137 foetuses genotyped as *Arrdc3*^*−/−*^. E18.5/19.5 pups also obeyed Mendelian ratios for each *Arrdc3* genotype (X^2^ = 1.511, df = 2, *p* > 0.05 (*p* = 0.470)). Expected numbers are marked with a red E. **C** E19.5 *Arrdc3*^*−/−*^ pups (*n* = 7 animals examined across 3 litters) displayed numerous gross morphological defects, though none were completely penetrant. **D** Underdeveloped eyes were observed in some *Arrdc3*^*−/−*^ (and *Arrdc3*^*+/−*^) animals. In this stronger example, the right eye of mouse 470.3 was not externally visible. **E** An omphalocele observed in *Arrdc3*^*−/−*^ mouse 468.3. **F** Haemorrhaging was observed on the exterior of some *Arrdc3*^*−/−*^ animals and in some internal organs, including the surface of the thymus in *Arrdc3*^*−/−*^ mouse 472.5. **G** Many *Arrdc3*^*−/−*^ pups had breathing difficulties, including mouse 468.2, which also displayed additional morphological defects, including subcutaneous oedema which included serous fluid and blood.

To explore the role of *Arrdc3* in vivo, we generated *Arrdc3* knockout mice by deleting 7 of 8 *Arrdc3* exons (Fig. 3A). After obtaining a stable colony of *Arrdc3*^*+/−*^ mice, we found that inter-crossing these mice did not yield viable *Arrdc3*^*−/−*^ adult offspring, as has been previously observed [20, 35]. Inter-crosses between *Arrdc3*^*+/−*^ animals revealed a statistically significant difference between the observed and expected numbers of *Arrdc3*^*−/−*^ animals at weaning (Fig. 3B). To determine the developmental stage when *Arrdc3*^*−/−*^ animals die, genotypes were assessed at embryonic day 14.5 (E14.5) after timed inter-crosses of *Arrdc3*^*+/−*^ mice, which revealed expected Mendelian ratios (Fig. 3B). We next assessed the foetal genotypes at E18.5/19.5, after timed inter-crosses of *Arrdc3*^*+/−*^ mice, by administering progesterone to pregnant females on E17.5 and E18.5, preventing labour. This allowed for a Caesarean section to be carried out to deliver the pups at E18.5/19.5. Genotyping of the E18.5/19.5 pups across 20 separate litters showed all genotypes were in line with Mendelian ratios (Fig. 3B). This reveals the *Arrdc3* loss-induced lethality likely occurs during or soon after birth (perinatally).

To identify abnormalities that might be contributing to this lethality, we carried out a full assessment of E19.5 pups (*n* = 21 animals examined across 3 litters) (File S1). There was a slight, but non-significant, trend towards *Arrdc3*^*+/−*^ and *Arrdc3*^*−/−*^ animals weighing more than their wild-type littermates (data not shown). Notably, we observed a number of incompletely penetrant developmental abnormalities in the *Arrdc3*^*−/−*^ pups (Fig. 3C), including: underdeveloped eyes (also seen in some *Arrdc3*^*+/−*^ animals) (Fig. 3C, D), external and internal haemorrhaging, manifesting, for example, as small areas of haemorrhage on the surface of the thymus (Fig. 3C, F), liver discolouration (Fig. 3C), and one *Arrdc3*^*−/−*^ animal presented with an omphalocele (Fig. 3C, E). Some *Arrdc3*^*−/−*^ pups had breathing difficulties, with one of these (pup 468.2) also displaying subcutaneous oedema composed of serous fluid and blood (Fig. 3C, G). We hypothesised that heart defects might contribute to the mortality of *Arrdc3*^*−/−*^ pups, given the lack of consistently lethal external morbidities. Histological sections of the hearts revealed some *Arrdc3*^*−/−*^ pups (*n* = 2/7) exhibited ventricular-septal defects. These defects varied in severity, with one *Arrdc3*^*−/−*^ animal missing gross internal ventricle structure (compare Figures S5A + B, demonstrating *Arrdc3*^*+/+*^ and *Arrdc3*^*+/−*^ hearts, to S5C, demonstrating a disrupted *Arrdc3*^*−/−*^ heart), while another *Arrdc3*^*−/−*^ animal had a very small ventricular-septal defect (Figure S5D). These findings indicate that a range of developmental defects may contribute to the perinatal lethality of *Arrdc3*^*−/−*^ mice.

### *Arrdc3* has no role in TRP53-mediated cell cycle arrest or apoptosis in primary non-transformed cells

Considering the requirement of ARRDC3 in development, we next investigated whether ARRDC3 plays a role in the survival and growth of primary tissues. We first examined murine embryonic fibroblasts (MEFs) derived from *Arrdc3*^*+/+*^ and *Arrdc3*^*−/−*^ E14.5 foetuses. Gene expression analysis via qRT-PCR was used to assess *Arrdc3* expression in MEFs after treatment with nutlin-3a or etoposide. As expected, *Arrdc3*^*−/−*^ MEFs had entirely lost *Arrdc3* expression (Figure S6A), but in wild-type (wt) MEFs we observed that *Arrdc3* expression was relatively weakly induced (~2-fold induction) in response to both drugs (Figure S6B). While considerably smaller than the responses observed in *Eμ-Myc* lymphoma cells (Figs. 2A, S3), the similarly reduced levels of induction of known TRP53-target control genes (*Pmaip1*, *Bbc3*, *Cdkn1a*) suggest that MEFs are overall less sensitive to TRP53-activating stimuli than *Eµ-Myc* lymphoma cells. We next examined the cell cycle behaviour of MEFs after treatment with nutlin-3a, which revealed the expected reduction in numbers of cells in S phase and increased numbers of cells in G_1_ phase, but no significant differences were evident between the two genotypes (Figure S6C). Finally, we assessed MEF viability after treatment with different concentrations of nutlin-3a or etoposide. Each treatment resulted in a noticeable reduction in MEF viability, but no significant differences were observed between the two genotypes (Figure S6D).

We next examined the role of *Arrdc3* in the development of primary haematopoietic cells. To enable this, we used the foetal liver cells of E14.5 *Arrdc3*^*+/+*^ and *Arrdc3*^*−/−*^ foetuses to perform haematopoietic reconstitutions, injecting these cells into lethally irradiated recipient wild-type congenic mice. After 10 weeks we harvested the bone marrow, spleen, and thymus and assessed the proportions and numbers of different haematopoietic cell types in these tissues by flow cytometry. Examining B cell development in the bone marrow did not reveal any differences between the *Arrdc3*^*+/+*^ (i.e. wt) *vs Arrdc3*^*−/−*^ reconstituted mice (Figure S7A). Similarly, in both genotypes, follicular and marginal zone B cells from the spleen were roughly equal in number (Figure S7B), as were T lymphoid cells of the major stages of differentiation (as defined by expression of CD4 and CD8) in the thymus (Figure S7C). We also examined the responses over time of cultured bone marrow-derived B cells and thymocytes from *Arrdc3*^*+/+*^ and *Arrdc3*^*−/−*^ reconstituted mice to treatment with different doses of nutlin-3a (Figure S7D). Like in MEFs, we found no differences in the viability of these cells at any timepoint between the two genotypes. Lastly, qRT-PCR was used to evaluate the expression of *Arrdc3* and *Cdkn1a* (*p21*, as a TRP53 target control) in splenic B cells and thymocytes from *Arrdc3*^*+/+*^ and *Arrdc3*^*−/−*^ reconstituted mice. In both B and T cells, *Arrdc3* expression was increased in the nutlin-3a treated *Arrdc3*^*+/+*^ cells but, as expected, absent in the *Arrdc3*^*−/−*^ samples, whereas *Cdkn1a* expression was strongly induced after treatment with nutlin-3a in cells from both genotypes (Figure S7E). The extent of *Arrdc3* induction in B cells was less pronounced than in T cells after 24 h of treatment with nutlin-3a (~2-fold *vs* ~10-fold) (Figure S7E).

While *Arrdc3* is required for normal embryonic development, it does not have a major role in haematopoiesis, or in the response of lymphoid cells or MEFs to anti-cancer agents that activate TRP53.

### The absence of *Arrdc3* markedly accelerates lymphoma development in *Eμ-Myc* transgenic mice

**Fig. 4:**
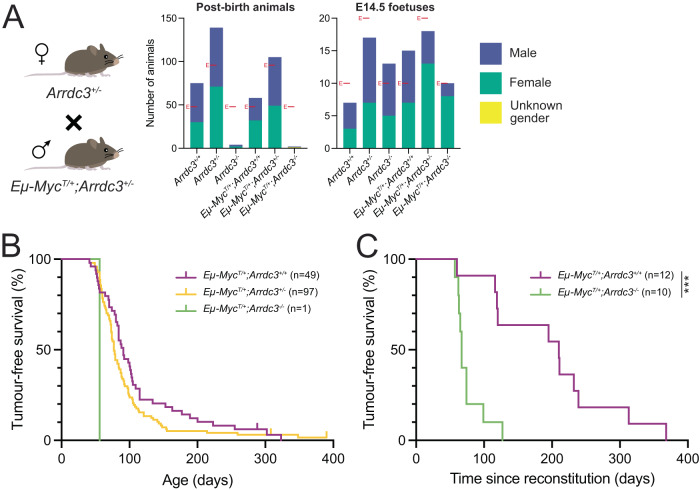
Loss of *Arrdc3* markedly accelerated MYC-driven lymphomagenesis in mice. **A** Offspring of inter-crosses between *Arrdc3*^*+/−*^ and *Eμ-Myc*^*T/+*^*;Arrdc3*^*+/−*^ mice do not obey Mendelian ratios post-birth (X^2^ = 122.11, df = 5, *p* < 0.05 (*p* < 1 × 10^−15^)). By contrast, E14.5 foetuses generated from such inter-crosses were seen to obey Mendelian ratios (X^2^ = 4.95, df = 5, *p* > 0.05 (*p* = 0.422)). Expected numbers are marked with a red E. **B** Kaplan–Meier survival curve of *Eμ-Myc*^*T/+*^;*Arrdc3*^*+/+*^ mice (median survival = 91 days), *Eμ-Myc*^*T/+*^;*Arrdc3*^*+/−*^ mice (median survival = 77 days), and only one *Eμ-Myc*^*T/+*^;*Arrdc3*^*−/−*^ mouse that survived the developmental perinatal lethality. The absence of one allele of *Arrdc3* slightly, but not significantly, accelerated lymphoma development in *Eμ-Myc* mice (Mantel-Cox test, df = 1, *p* > 0.05 (*p* = 0.0686)). **C** Kaplan–Meier lymphoma-free survival curve of lethally irradiated recipient mice that had been transplanted with *Eμ-Myc*^*T/+*^*;Arrdc3*^*+/+*^ or *Eμ-Myc*^*T/+*^*;Arrdc3*^*−/−*^ foetal liver cells. We observed a statistically significant acceleration of lymphoma development in mice that had been transplanted with *Eμ-Myc*^*T/+*^*;Arrdc3*^*−/−*^ foetal liver cells (median survival = 67 days) compared to control mice that had been transplanted with *Eμ-Myc*^*T/+*^*;Arrdc3*^*+/+*^ foetal liver cells (median survival = 210 days) (Mantel-Cox test, df = 1, X^2^ = 13.22, *p* < 0.001 (*p* = 0.000276)).

Having found that loss of *Arrdc3* confers a competitive advantage in malignant *Eμ-Myc* lymphoma cells, and having generated an *Arrdc3* knockout mouse model, we next investigated whether *Arrdc3* might impact MYC-driven lymphoma development in vivo. To this end, we inter-crossed *Eµ-Myc*^*T/+*^*;Arrdc3*^*+/−*^ male mice with *Arrdc3*^*+/−*^ female mice. Genotyping the offspring of these crosses revealed that, for mice with or without an *Eμ-Myc* transgene, *Arrdc3*^*−/−*^ mice were significantly underrepresented at the adult stage (Fig. 4A). We then monitored those mice possessing an *Eµ-Myc* transgene to determine the impact of *Arrdc3* loss on MYC-driven lymphoma development. Surprisingly, one *Eμ-Myc*^*T/+*^*;Arrdc3*^*−/−*^ animal survived post-weaning but had to be sacrificed due to lymphoma at 56 days (Fig. 4B). Assessing the tumour-free survival of the other genotypes, we observed a slight but non-significant decrease in tumour latency in *Eμ-Myc*^*T/+*^*;Arrdc3*^*+/−*^ animals (median survival = 77 days) compared to the *Eμ-Myc*^*T/+*^*;Arrdc3*^*+/+*^ control mice (median survival = 91 days) (Mantel-Cox test, df = 1, X^2^ = 2.981, *p* > 0.05 (*p* = 0.0842)) (Fig. 4B). Stratifying the mice by gender did not reveal any additional variation between genotypes.

As we were unable to obtain more than one *Eμ-Myc*^*T/+*^*;Arrdc3*^*−/−*^ adult, we turned to the process of haematopoietic reconstitution. At E14.5 we observed the genotypes fell into expected Mendelian ratios (Fig. 4A), and therefore we were able to use both *Eμ-Myc*^*T/+*^*;Arrdc3*^*+/+*^ and *Eμ-Myc*^*T/+*^*;Arrdc3*^*−/−*^ foetal liver cells to reconstitute lethally irradiated recipient mice. These recipients were then monitored for lymphoma development. We observed a remarkable (statistically significant) decrease in tumour latency in mice reconstituted with *Eμ-Myc*^*T/+*^*;Arrdc3*^*−/−*^ foetal liver cells (median survival = 67 days) compared to recipients reconstituted with *Eμ-Myc*^*T/+*^*;Arrdc3*^*+/+*^ foetal liver cells (median survival = 210 days) (Mantel-Cox test, df = 1, X^2^ = 13.22, *p* < 0.001 (*p* = 0.000276)) (Fig. 4C). Examining the peripheral blood content of the lymphoma burdened mice at sacrifice revealed no clear differences between the two genotypes in their cellular makeup (Figure S8A). Similarly, organ weights did not reveal any clear differences between the two genotypes and hence severity of lymphomatous disease (Figure S8B). Interestingly, immunophenotyping of the malignant cells derived from the spleens of the reconstituted mice illustrated some differences between the genotypes. The *Eμ-Myc*^*T/+*^*;Arrdc3*^*−/−*^ lymphomas were more immature in origin (>60% tumours were majority B220+/IgD-/IgM- pro-B/pre-B) compared to the *Eμ-Myc*^*T/+*^*;Arrdc*^*+/+*^ lymphomas (>60% tumours were majority B220+/IgD-/IgM+ immature B) (Figure S8C).

Collectively, these findings demonstrate *Arrdc3* loss leads to a marked acceleration of MYC-driven lymphoma development.

## Discussion

In this study, we have identified a novel role for ARRDC3 as a mediator of TRP53-mediated tumour suppression in *Eμ-Myc*-driven lymphomagenesis. ARRDC3 is one of 10 arrestin family members in mammals, with research showing there is at least partial overlap between the functionality of these proteins, and some level of physical association between them too, though the significance of the association is not completely understood [18, 20]. Likely the best characterised function for ARRDC3, shared with the β-arrestins, is as a regulator of receptor-mediated signalling. In this role, ARRDC3 wears multiple hats, as while it has been shown to promote G-protein coupled receptor (GPCR) signalling via the beta-2 adrenergic receptor [21], it can also negatively regulate various signalling pathways. One such example is insulin signalling, where ARRDC3 suppresses phosphorylation of FOXO1 by AKT [36]. Another GPCR example is PAR1, where ARRDC3 suppresses Hippo signalling by binding and inhibiting pathway effectors YAP and TAZ, a function that also appears to be conserved in tissues of the vinegar fly, *Drosophila melanogaster* [16, 26, 37]. A number of these roles occur at the endosomal level, with α-arrestins 1 and 4 also having been shown to act at the level of extracellular vesicles [38, 39], suggesting a general importance for α-arrestins in cellular trafficking processes. Mechanistically, clues regarding ARRDC3 functions can be found in its structure—specifically, two PPxY domains (shared by most α-arrestins) facilitate binding to WW domain-containing proteins, such as various NEDD4-family E3 ubiquitin ligases, including ITCH, NEDD4, NEDD4L, WWP1, and WWP2, some of which have been shown to contribute to ARRDC3-mediated regulation of molecular signalling [17–20, 25, 40]. Despite this impressive body of work, and its discovery over a decade ago [41], ARRDC3 function has still only begun to be characterised.

No role for ARRDC3 as a mediator of the TP53 tumour suppressive pathway has previously been identified. However, interestingly, better characterised arrestin family members, such as the β-arrestins (arrestin beta 1 and arrestin beta 2 (ARRB1/2)), have been identified as either promoters or suppressors of apoptosis [42]. Due to the nature of our identification of *Arrdc3* in screens involving activation of TRP53, the most intriguing connection is the ability of ARRB2 to bind MDM2, the E3 ubiquitin ligase which is primarily responsible for the degradation of TP53 [43]. Indeed, in this role, ARRB2 activity appears to be partially reliant on GPCR signalling, an increase of which correlated with ARRB2-MDM2 binding strength [43]. While a similar relationship between MDM2 and ARRDC3 would line up well with our observations of a role for ARRDC3 in TP53-mediated tumour suppression, no evidence exists for ARRDC3 regulating MDM2. However, it is noteworthy that NEDD4, WWP1, and WWP2, some of the HECT E3 ubiquitin ligases thought to interact physically with ARRDC3, are also thought to act as regulators of MDM2 and/or TP53 [44–46]. As such, exploration of the proteome surrounding ARRDC3, particularly in malignant cells, would help inform our understanding of the role of ARRDC3.

At least two other groups have previously generated *Arrdc3* knockout mice via independent gene trap insertions, as opposed to our CRISPR/Cas9 approach [20, 35]. Interestingly, while we observed perinatal lethality in *Arrdc3*^*−/−*^ animals, Patwari et al. observed ~7% homozygous knockout animals surviving to weaning [35], and while Shea et al. observed no surviving homozygous knockout animals, they were able to partially rescue homozygous knockout lethality by providing the breeding mice with a high-fat diet [20]. While varying diet is not something we tested, we did observe significant morphological defects in E19.5 animals (Fig. 3), something other groups did not examine. Particularly noteworthy are the heart defects we observed which, while incompletely penetrant (Figure S5), generate some intriguing connections with other literature. For example, in humans, *ARRDC3* copy number variation has been linked to congenital heart defects [47]. In *Drosophila melanogaster*, an unnamed *ARRDC3* orthologue (*CG1105*) was the 10th strongest hit in a 7000+ gene RNAi screen for regulators of heart function [48]. ARRDC3 variants have also been associated with congestive heart failure in cattle [49]. In the context of human malignancies, analysis of 5989 patient samples of haematopoietic cancers using the cBioPortal database showed no correlation between ARRDC3 mutation (loss/amplification) with either MYC amplification or TP53 loss [50–52]. However, expanding this search to include all types of cancers, across 69223 patient samples, the database indicated ARRDC3 mutation (loss/amplification) co-occurred significantly (two-sided Fischer exact tests, each *p* < 0.001) with both MYC amplificiation and TP53 loss. These results suggest there may be an unappreciated role for ARRDC3 in human cancers and, coupled with our phenotypic observations and the literature studying ARRDC3 in a highly diverse set of contexts, there is some level of universal importance for ARRDC3.

In summary, we have characterised a role for ARRDC3 in regulating the process of TP53-mediated suppression of *Eμ-Myc*-driven lymphomagenesis. This is a novel role for this relatively understudied member of the arrestin gene family. Further investigations into ARRDC3 and related arrestin family members, and in particular their relationship to TRP53, are certainly warranted, and may uncover new therapeutic avenues or key regulatory avenues that could bypass defects in TP53 for cancer therapy.

## Materials and methods

### CRISPR/Cas9 screening with nutlin-3a

Once stably transfected AF47A-Cas9-Yusa *Eμ-Myc* lymphoma cells were generated (see Supplementary Data), input samples were collected for DNA. 16 × 10^6^ AF47A-Cas9-Yusa lymphoma cells were then plated into T125 flasks in quadruplicate, and each replicate treated with nutlin-3a (10 μM, Cayman Chemical #18585) or DMSO (vehicle control; Sigma-Aldrich #D4540) for 24 h. From the treated samples, the remaining live cells (propidium iodide (PI)-negative) were sorted via FACS, and samples taken for DNA extraction.

### CRISPR screen sequencing and analysis

DNA was extracted from all samples using the DNeasy Blood and Tissue kit (QIAGEN #69506). Vector indexing was performed by single-step PCR using established primers [29] which contain overhangs to enable preparation of an Illumina sequencing library. Indexed samples were then pooled, DNA clean up performed using Ampure XP Beads (Beckman Coulter #A63880), and pools prepared for sequencing on a MiSeq (Illumina) machine according to the manufacturer’s instructions. MAGeCK v0.4 and v0.5 [53] was used to rank sgRNA enrichment within the different treatment samples.

### Mouse husbandry and generation of *Arrdc3* knockout mice

*Eµ-Myc* transgenic mice (which are always kept as heterozygotes) have been reported [13] and were maintained on a C57BL/6 background. See supplementary materials for additional information.

To generate the *Arrdc3* knockout mouse strain, exons 1-7 of *Arrdc3* (mouse chromosome 13) were deleted in C57BL/6J fertilised oocytes by CRISPR editing using two sgRNAs with the sequences 5′-GAGACTACTAGGTGACGGGAAGG-3′ and 5′-AGCCATCCTCATCGACTACAGGG-3′ following established protocols [32]. Genotyping primers and expected sizes are listed in Table S2. To minimise CRISPR off-targets, mice were backcrossed twice to C57BL/6 mice, and frequently outcrossed to *Eµ-Myc* mice. Deletion of the targeted region in the *Arrdc3* gene was confirmed by NGS.

### Supplementary information

Supplemental Figures

Supplementary Figure Legends

Supplementary Methods, File Legends, Tables

Supplemental File 1

Supplemental File 2_MAGeCK

Reproducibility Checklist

## Data Availability

CRISPR screen data is available in Supplementary File 2. All other raw data is available from the corresponding author upon request.

## References

[1] Lossi L (2022). The concept of intrinsic versus extrinsic apoptosis. Biochem J.

[2] Nakano K, Vousden KH (2001). PUMA, a novel proapoptotic gene, is induced by p53. Mol Cell.

[3] Oda E, Ohki R, Murasawa H, Nemoto J, Shibue T, Yamashita T (2000). Noxa, a BH3-only member of the Bcl-2 family and candidate mediator of p53-induced apoptosis. Science.

[4] Tong T, Ji J, Jin S, Li X, Fan W, Song Y (2005). Gadd45a expression induces Bim dissociation from the cytoskeleton and translocation to mitochondria. Mol Cell Biol.

[5] Yu J, Zhang L, Hwang PM, Kinzler KW, Vogelstein B (2001). PUMA induces the rapid apoptosis of colorectal cancer cells. Mol Cell.

[6] Singh R, Letai A, Sarosiek K (2019). Regulation of apoptosis in health and disease: the balancing act of BCL-2 family proteins. Nat Rev Mol Cell Biol.

[7] Kastenhuber ER, Lowe SW (2017). Putting p53 in context. Cell.

[8] Cao X, Hou J, An Q, Assaraf YG, Wang X (2020). Towards the overcoming of anticancer drug resistance mediated by p53 mutations. Drug Resist Updat.

[9] Harper JW, Adami GR, Wei N, Keyomarsi K, Elledge SJ (1993). The p21 Cdk-interacting protein Cip1 is a potent inhibitor of G1 cyclin-dependent kinases. Cell.

[10] Valente LJ, Gray DH, Michalak EM, Pinon-Hofbauer J, Egle A, Scott CL (2013). p53 efficiently suppresses tumor development in the complete absence of its cell-cycle inhibitory and proapoptotic effectors p21, Puma, and Noxa. Cell Rep.

[11] Donehower LA, Harvey M, Slagle BL, McArthur MJ, Montgomery CA, Butel JS (1992). Mice deficient for p53 are developmentally normal but susceptible to spontaneous tumours. Nature.

[12] Janic A, Valente LJ, Wakefield MJ, Di Stefano L, Milla L, Wilcox S (2018). DNA repair processes are critical mediators of p53-dependent tumor suppression. Nat Med.

[13] Adams JM, Harris AW, Pinkert CA, Corcoran LM, Alexander WS, Cory S (1985). The c-myc oncogene driven by immunoglobulin enhancers induces lymphoid malignancy in transgenic mice. Nature.

[14] Puca L, Brou C (2014). Α-arrestins—new players in Notch and GPCR signaling pathways in mammals. J Cell Sci.

[15] Arakaki AKS, Pan WA, Lin H, Trejo J (2018). The α-arrestin ARRDC3 suppresses breast carcinoma invasion by regulating G protein-coupled receptor lysosomal sorting and signaling. J Biol Chem.

[16] Arakaki AKS, Pan WA, Wedegaertner H, Roca-Mercado I, Chinn L, Gujral TS, et al. α-Arrestin ARRDC3 tumor suppressor function is linked to GPCR-induced TAZ activation and breast cancer metastasis. J Cell Sci. 2021;134:1–15. 10.1242/jcs.254888 PMC8084569 33722977

[17] Dores MR, Lin H, N JG, Mendez F, Trejo J (2015). The α-arrestin ARRDC3 mediates ALIX ubiquitination and G protein-coupled receptor lysosomal sorting. Mol Biol Cell.

[18] Han SO, Kommaddi RP, Shenoy SK (2013). Distinct roles for β-arrestin2 and arrestin-domain-containing proteins in β2 adrenergic receptor trafficking. EMBO Rep.

[19] Nabhan JF, Pan H, Lu Q (2010). Arrestin domain-containing protein 3 recruits the NEDD4 E3 ligase to mediate ubiquitination of the beta2-adrenergic receptor. EMBO Rep.

[20] Shea FF, Rowell JL, Li Y, Chang TH, Alvarez CE (2012). Mammalian α arrestins link activated seven transmembrane receptors to Nedd4 family e3 ubiquitin ligases and interact with β arrestins. PLoS ONE.

[21] Tian X, Irannejad R, Bowman SL, Du Y, Puthenveedu MA, von Zastrow M (2016). The α-arrestin ARRDC3 regulates the endosomal residence time and intracellular signaling of the β2-adrenergic receptor. J Biol Chem.

[22] Draheim KM, Chen HB, Tao Q, Moore N, Roche M, Lyle S (2010). ARRDC3 suppresses breast cancer progression by negatively regulating integrin beta4. Oncogene.

[23] Lei D, Deng N, Wang S, Huang J, Fan C (2020). Upregulated ARRDC3 limits trophoblast cell invasion and tube formation and is associated with preeclampsia. Placenta.

[24] Leonard MK, Puts GS, Pamidimukkala N, Adhikary G, Xu Y, Kwok E (2021). Comprehensive molecular profiling of UV-induced metastatic melanoma in Nme1/Nme2-deficient mice reveals novel markers of survival in human patients. Oncogene.

[25] Soung YH, Kashyap T, Nguyen T, Yadav G, Chang H, Landesman Y (2017). Selective Inhibitors of Nuclear Export (SINE) compounds block proliferation and migration of triple negative breast cancer cells by restoring expression of ARRDC3. Oncotarget.

[26] Xiao J, Shi Q, Li W, Mu X, Peng J, Li M (2018). ARRDC1 and ARRDC3 act as tumor suppressors in renal cell carcinoma by facilitating YAP1 degradation. Am J Cancer Res.

[27] Zhang B, Wu F, Li P, Li H. ARRDC3 inhibits liver fibrosis and epithelial-to-mesenchymal transition via the ITGB4/PI3K/Akt signaling pathway. Immunopharmacol Immunotoxicol. 2022;45:1–12. 10.1080/08923973.2022.2128369 36154540

[28] Zheng Y, Lin ZY, Xie JJ, Jiang FN, Chen CJ, Li JX (2017). ARRDC3 inhibits the progression of human prostate cancer through ARRDC3-ITGβ4 pathway. Curr Mol Med.

[29] Koike-Yusa H, Li Y, Tan EP, Velasco-Herrera Mdel C, Yusa K (2014). Genome-wide recessive genetic screening in mammalian cells with a lentiviral CRISPR-guide RNA library. Nat Biotechnol.

[30] Valente LJ, Aubrey BJ, Herold MJ, Kelly GL, Happo L, Scott CL (2016). Therapeutic response to non-genotoxic activation of p53 by Nutlin3a is driven by PUMA-mediated apoptosis in lymphoma cells. Cell Rep.

[31] Thijssen R, Diepstraten ST, Moujalled D, Chew E, Flensburg C, Shi MX (2021). Intact TP-53 function is essential for sustaining durable responses to BH3-mimetic drugs in leukemias. Blood.

[32] Aubrey BJ, Kelly GL, Kueh AJ, Brennan MS, O’Connor L, Milla L (2015). An inducible lentiviral guide RNA platform enables the identification of tumor-essential genes and tumor-promoting mutations in vivo. Cell Rep.

[33] Vassilev LT, Vu BT, Graves B, Carvajal D, Podlaski F, Filipovic Z (2004). In vivo activation of the p53 pathway by small-molecule antagonists of MDM2. Science.

[34] Jaskulska A, Janecka AE, Gach-Janczak K. Thapsigargin-from traditional medicine to anticancer drug. Int J Mol Sci. 2020;22:1–12. 10.3390/ijms22010004 PMC7792614 33374919

[35] Patwari P, Emilsson V, Schadt EE, Chutkow WA, Lee S, Marsili A (2011). The arrestin domain-containing 3 protein regulates body mass and energy expenditure. Cell Metab.

[36] Batista TM, Dagdeviren S, Carroll SH, Cai W, Melnik VY, Noh HL (2020). Arrestin domain-containing 3 (Arrdc3) modulates insulin action and glucose metabolism in liver. Proc Natl Acad Sci USA.

[37] Shen X, Sun X, Sun B, Li T, Wu G, Li Y (2018). ARRDC3 suppresses colorectal cancer progression through destabilizing the oncoprotein YAP. FEBS Lett.

[38] Anand S, Foot N, Ang CS, Gembus KM, Keerthikumar S, Adda CG (2018). Arrestin-domain containing protein 1 (Arrdc1) regulates the protein cargo and release of extracellular vesicles. Proteomics.

[39] Mackenzie K, Foot NJ, Anand S, Dalton HE, Chaudhary N, Collins BM (2016). Regulation of the divalent metal ion transporter via membrane budding. Cell Discov.

[40] Qi S, O’Hayre M, Gutkind JS, Hurley JH (2014). Structural and biochemical basis for ubiquitin ligase recruitment by arrestin-related domain-containing protein-3 (ARRDC3). J Biol Chem.

[41] Oka S, Masutani H, Liu W, Horita H, Wang D, Kizaka-Kondoh S (2006). Thioredoxin-binding protein-2-like inducible membrane protein is a novel vitamin D3 and peroxisome proliferator-activated receptor (PPAR)gamma ligand target protein that regulates PPARgamma signaling. Endocrinology.

[42] Kook S, Gurevich VV, Gurevich EV (2014). Arrestins in apoptosis. Handb Exp Pharm.

[43] Wang P, Gao H, Ni Y, Wang B, Wu Y, Ji L (2003). Beta-arrestin 2 functions as a G-protein-coupled receptor-activated regulator of oncoprotein Mdm2. J Biol Chem.

[44] Che H, He W, Feng J, Dong W, Liu S, Chen T (2020). WWP2 ameliorates acute kidney injury by mediating p53 ubiquitylation and degradation. Cell Biochem Funct.

[45] Laine A, Ronai Z (2007). Regulation of p53 localization and transcription by the HECT domain E3 ligase WWP1. Oncogene.

[46] Xu C, Fan CD, Wang X (2015). Regulation of Mdm2 protein stability and the p53 response by NEDD4-1 E3 ligase. Oncogene.

[47] Derwińska K, Bartnik M, Wiśniowiecka-Kowalnik B, Jagła M, Rudziński A, Pietrzyk JJ (2012). Assessment of the role of copy-number variants in 150 patients with congenital heart defects. Med Wieku Rozwoj.

[48] Neely GG, Kuba K, Cammarato A, Isobe K, Amann S, Zhang L (2010). A global in vivo Drosophila RNAi screen identifies NOT3 as a conserved regulator of heart function. Cell.

[49] Heaton M, Harhay G, Bassett A, Clark H, Carlson J, Jobman E, et al. Association of ARRDC3 and NFIA variants with bovine congestive heart failure in feedlot cattle. F1000Research 2022;11:1–27. 10.12688/f1000research.109488.2 PMC11046187 38680232

[50] Cerami E, Gao J, Dogrusoz U, Gross BE, Sumer SO, Aksoy BA (2012). The cBio cancer genomics portal: an open platform for exploring multidimensional cancer genomics data. Cancer Discov.

[51] de Bruijn I, Kundra R, Mastrogiacomo B, Tran TN, Sikina L, Mazor T, et al. Analysis and visualization of longitudinal genomic and clinical data from the AACR project GENIE biopharma collaborative in cBioPortal. Cancer Res. 2023;83:3861–67. 10.1158/0008-5472.CAN-23-0816 PMC10690089 37668528

[52] Gao J, Aksoy BA, Dogrusoz U, Dresdner G, Gross B, Sumer SO (2013). Integrative analysis of complex cancer genomics and clinical profiles using the cBioPortal. Sci Signal.

[53] Li W, Xu H, Xiao T, Cong L, Love MI, Zhang F (2014). MAGeCK enables robust identification of essential genes from genome-scale CRISPR/Cas9 knockout screens. Genome Biol.

