# Supplementary material for: Genome-wide CRISPR screening identifies a role for ARRDC3 in TRP53-mediated responses
Source: Cell Death Differ. 2023 Dec 14;31(2):150–8. doi: 10.1038/s41418-023-01249-3 (PMC10850147; doi:10.1038/s41418-023-01249-3)

# Supplementary Figure 1

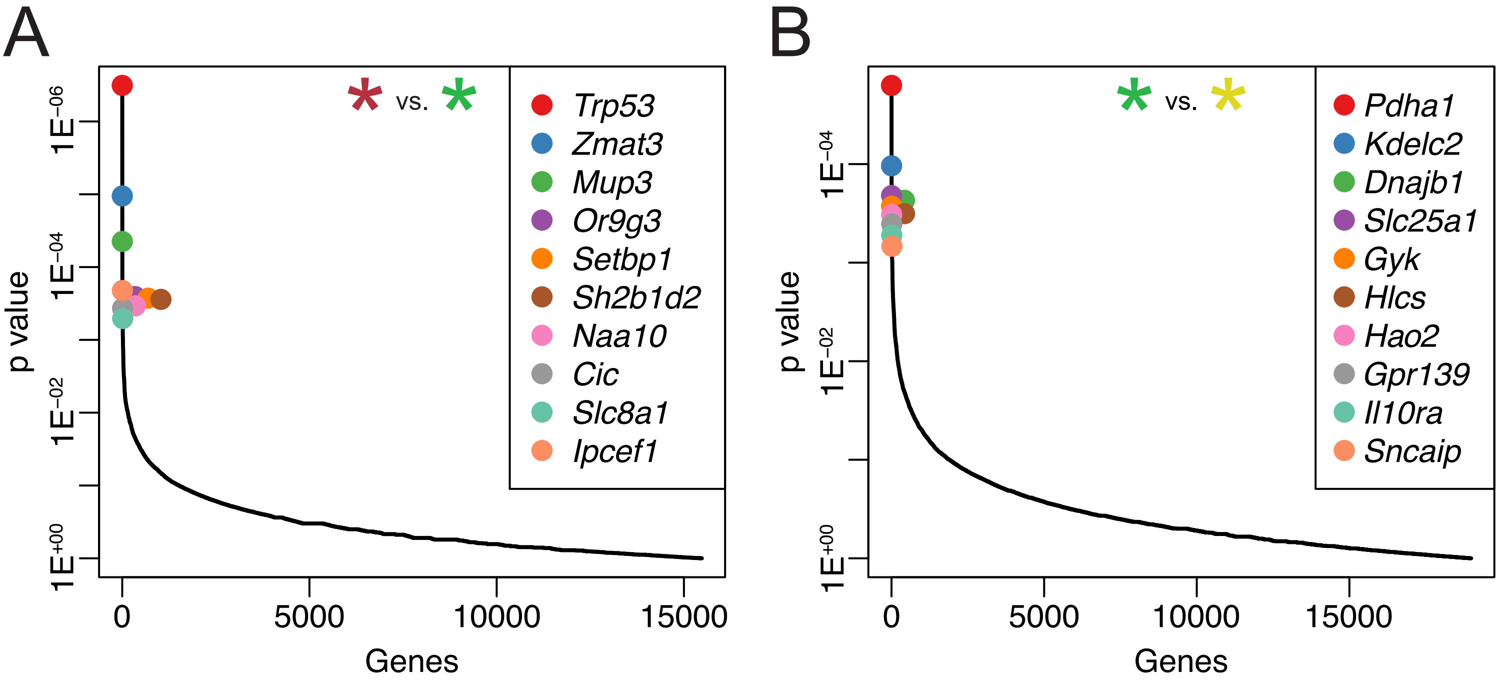

# Supplementary Figure 2

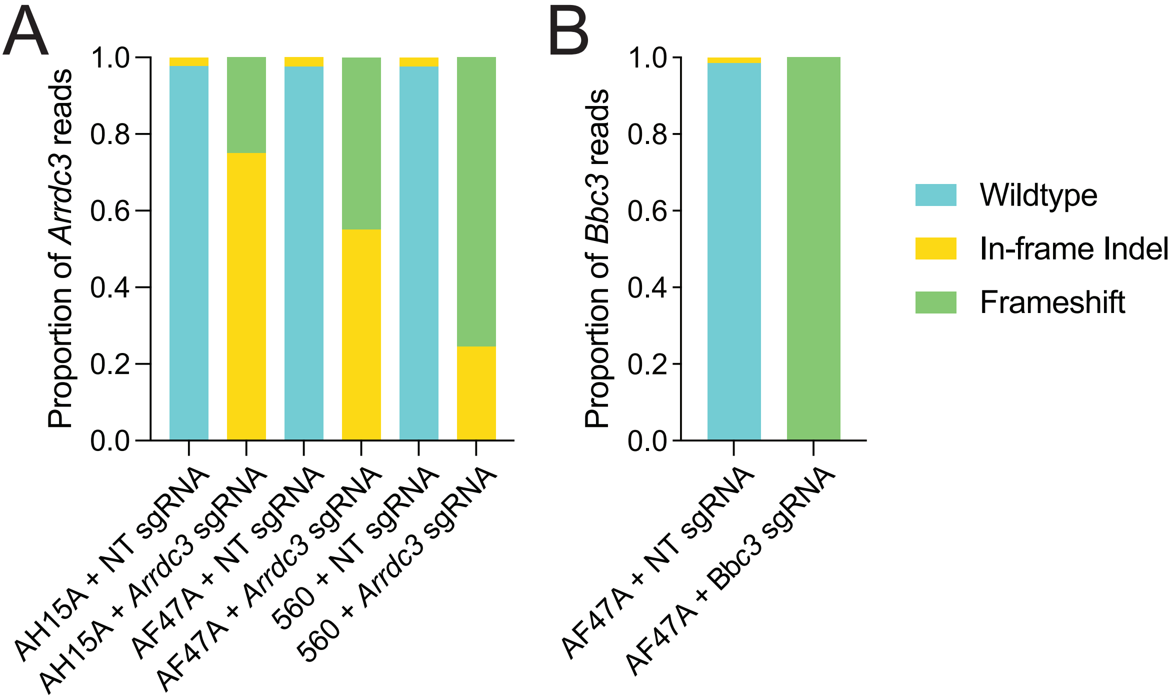

# Supplementary Figure 3

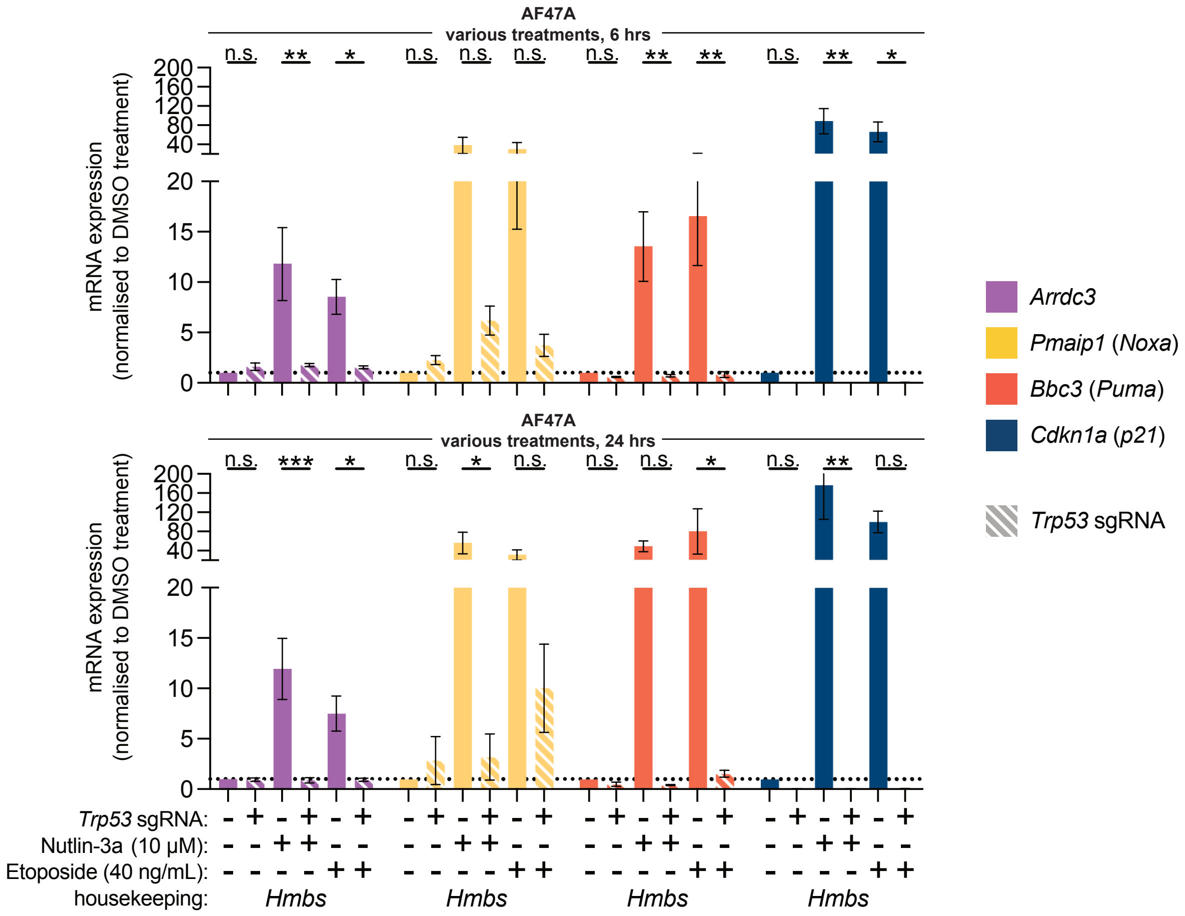

# Supplementary Figure 4

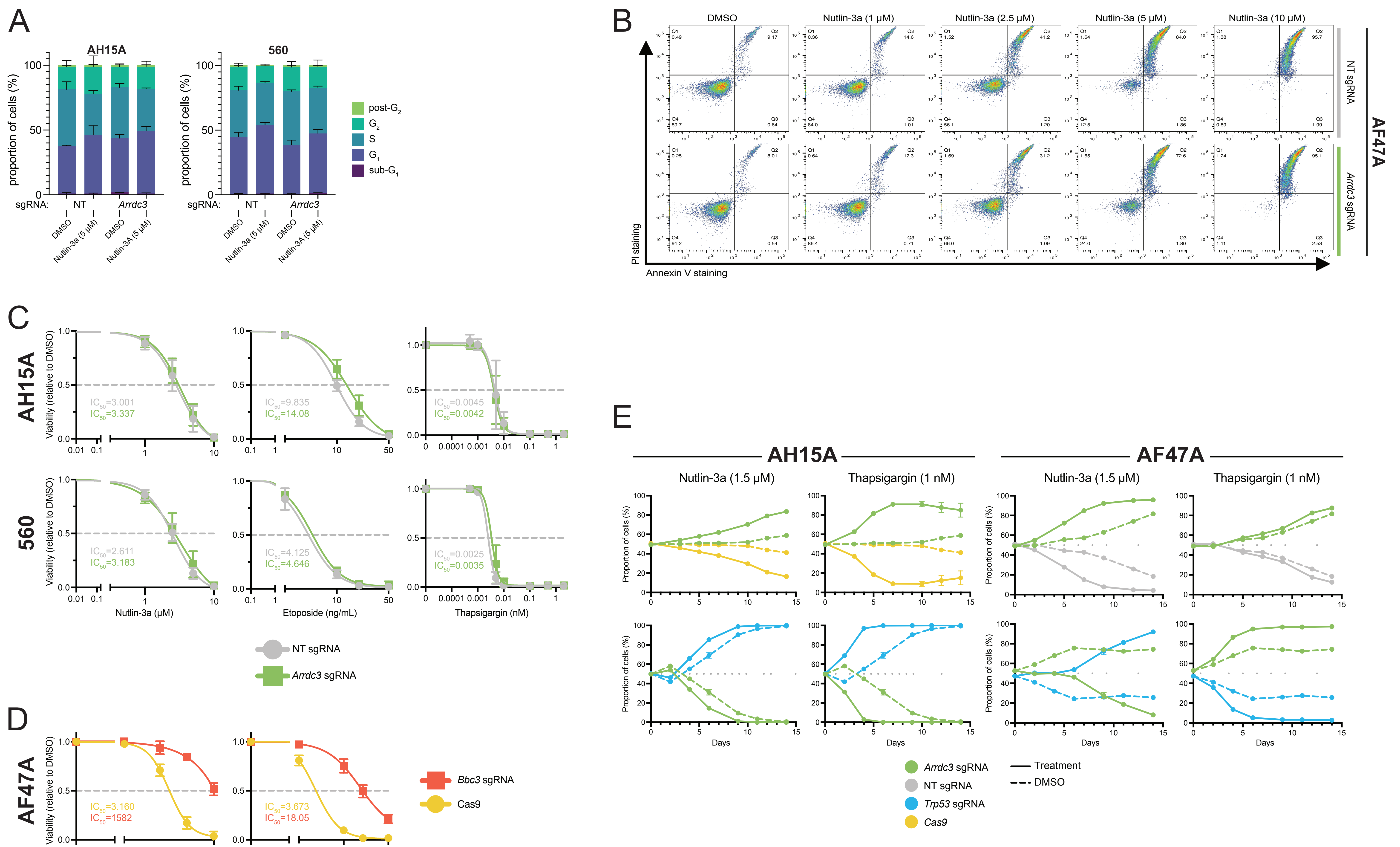

# Supplementary Figure 5

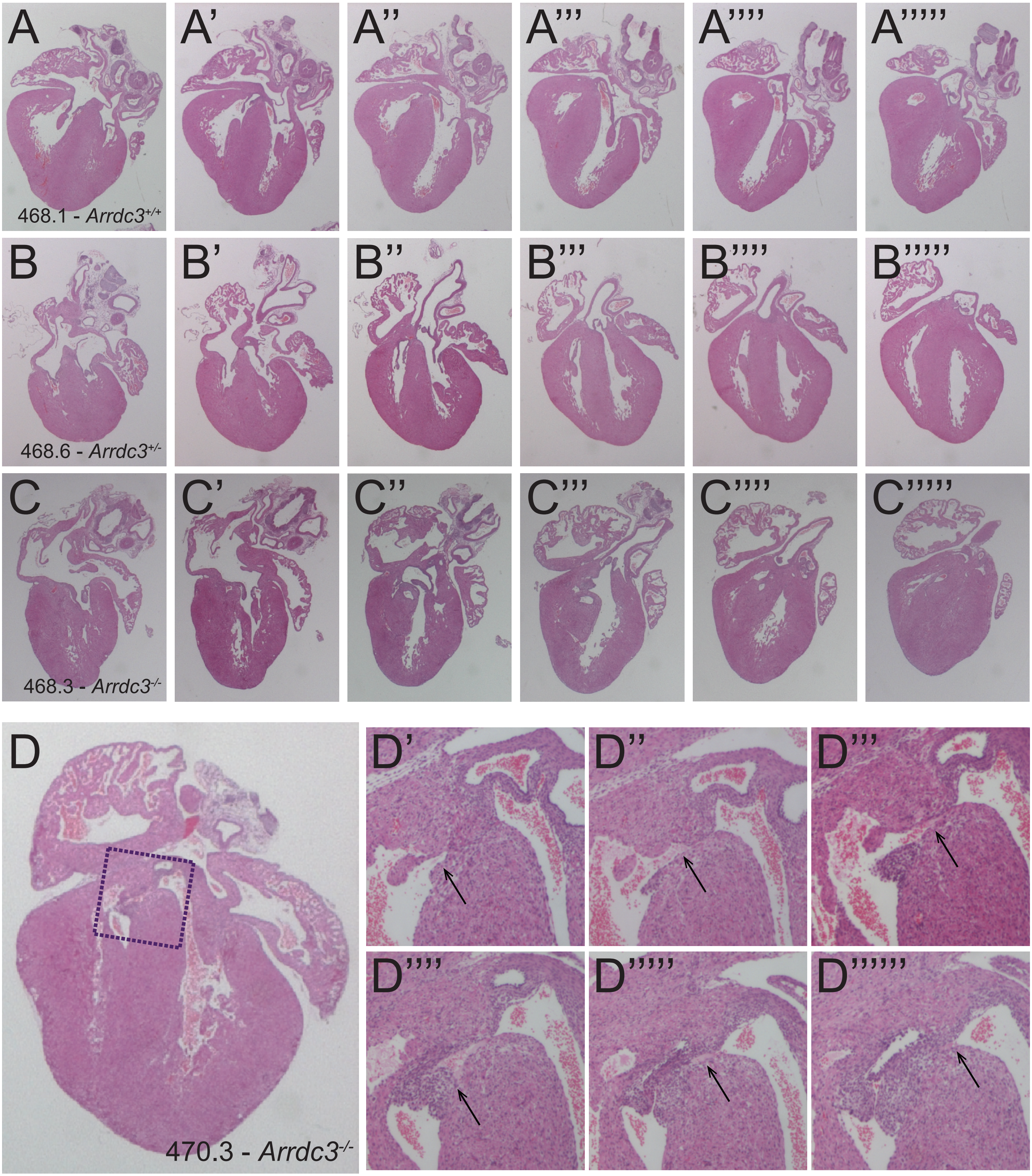

# Supplementary Figure 6

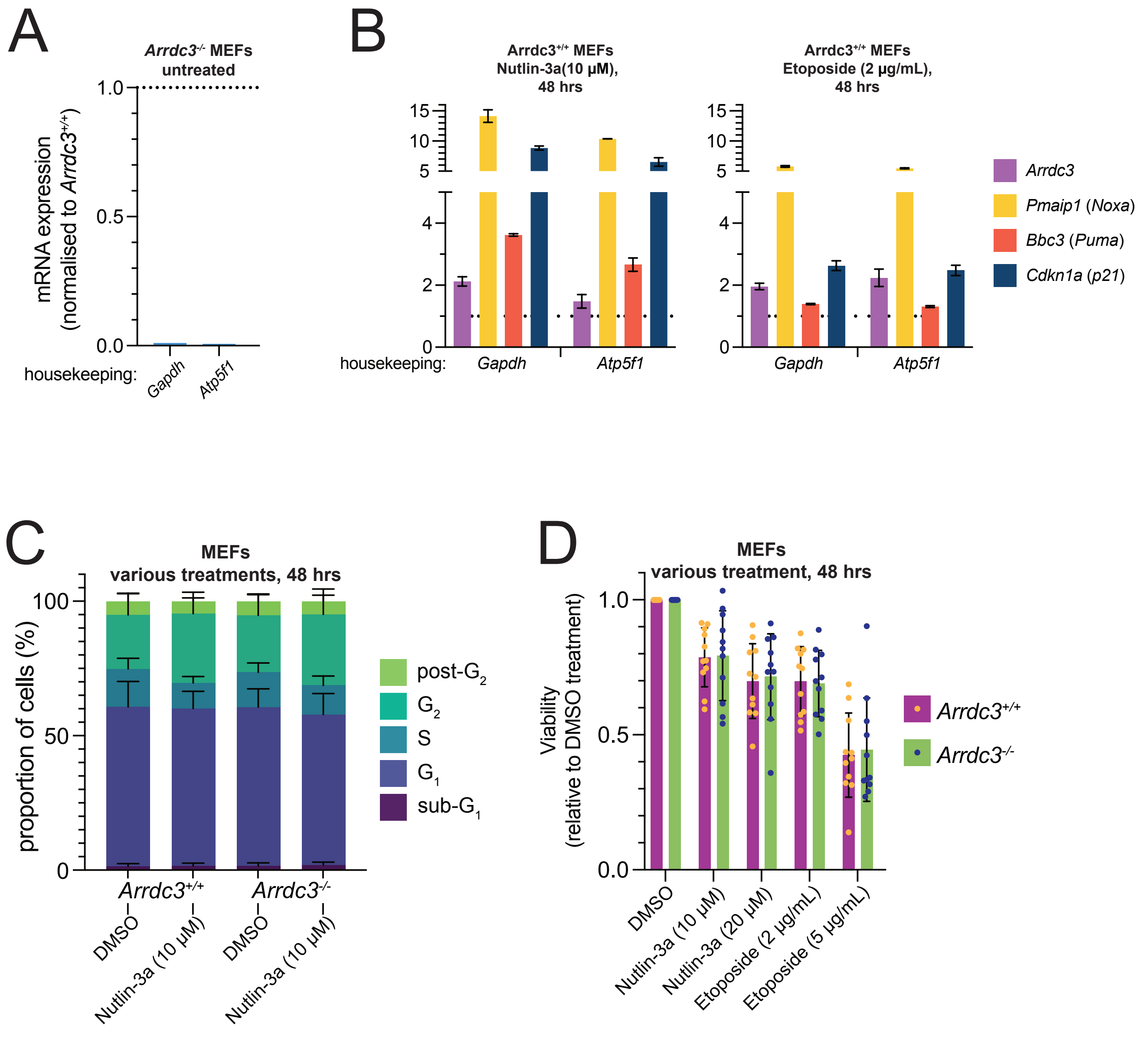

# Supplementary Figure 7

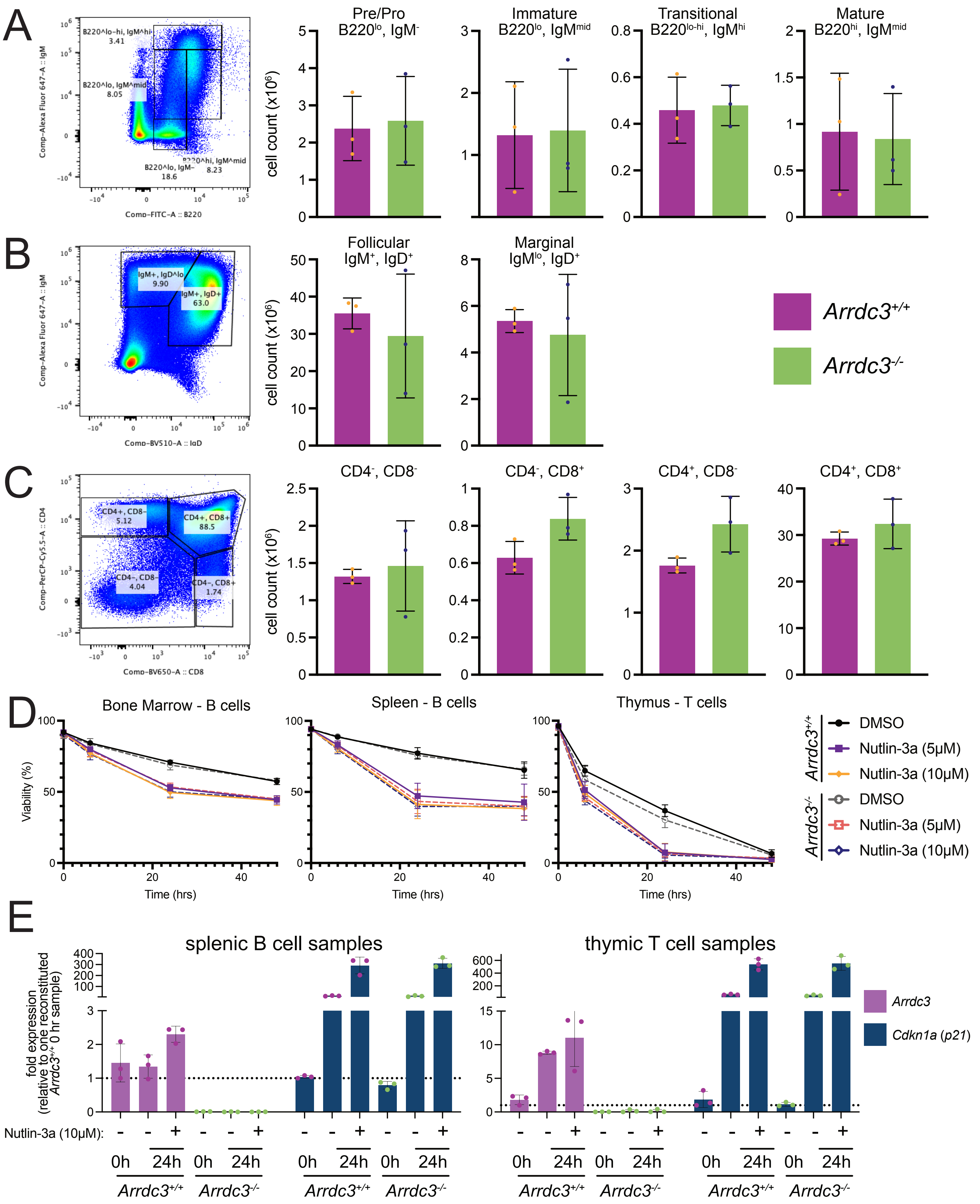

# Supplementary Figure 8

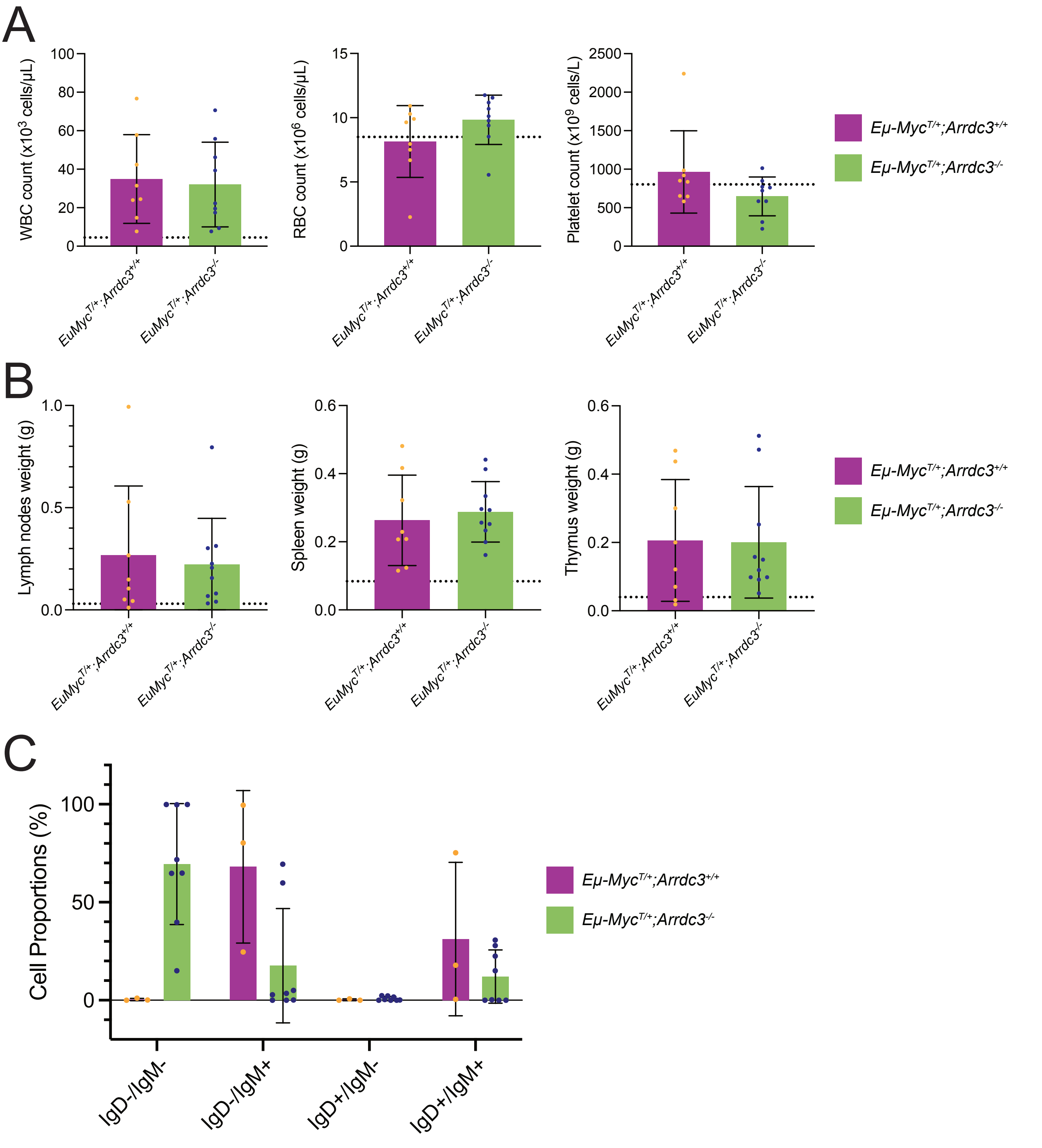

Supplement: Supplementary file 1 — Supplemental Figures [file 41418_2023_1249_MOESM1_ESM.pdf]
