# Supplementary material for: Genome-wide CRISPR screening identifies a role for ARRDC3 in TRP53-mediated responses
Source: Cell Death Differ. 2023 Dec 14;31(2):150–8. doi: 10.1038/s41418-023-01249-3 (PMC10850147; doi:10.1038/s41418-023-01249-3)
Supplement: Supplementary file 2 — Supplementary Figure Legends [file 41418_2023_1249_MOESM2_ESM.docx]

**Supplementary Figure 1. CRISPR screen results comparing DMSO with nutlin-3a treated *Eμ-Myc* lymphoma cell samples.** (A) Top hits from the CRISPR screen, comparing the *Eμ-Myc* lymphoma cells treated with DMSO (Figure 1A, green asterisk) with the *Eμ-Myc* lymphoma cells treated with nutlin-3a (Figure 1A, red asterisk). Using this comparison, *Arrdc3* is the 21^st^ top hit, likely due to the sgRNA(s) targeting *Arrdc3* being slightly enriched in the DMSO-treated samples. (B) Top hits from the CRISPR screen, comparing the untreated control cells (Figure 1A, yellow asterisk) with the cells treated with DMSO (Figure 1A, green asterisk). In this comparison, *Arrdc3* is not a hit, suggesting that even if loss of *Arrdc3* is selected for in DMSO-treated cells, it is not as strong as when nutlin-3a is present to activate TRP53 signalling.

**Supplementary Figure 2. Validation of *Arrdc3* gene editing by CRISPR/Cas9 in *Eμ-Myc* lymphoma cell lines.** (A) Next generation sequencing, in combination with bioinformatic analysis using CRISPR indel calculator software (<https://sarah-d.shinyapps.io/crispr-indel/>) revealed the CRISPR/Cas9 mediated knockout of *Arrdc3* in the AH15A, AF47A, and 560 *Eμ-Myc* lymphoma cell lines was highly efficient, with essentially no wild-type *Arrdc3* sequence being detected in any of these lymphoma cell lines. (B) An AF47A *Bbc3*/PUMA knockout cell line was also generated and validated via the same method, and returned similarly efficiency.

**Supplementary Figure 3. qRT-PCR analysis of the expression of TRP53 target genes in *Eμ-Myc* lymphoma cell lines using an alternative housekeeping gene.** Data from additional qRT-PCR analysis using *Hmbs* as the housekeeping gene, assessing the level of *Arrdc3* expression in AF47A *Eμ-Myc* lymphoma cells in response to treatment with nutlin-3a (10 μM) or etoposide (40 ng/mL) treatment for 6 or 24 h, relative to expression in DMSO-treated control lymphoma cells. Expression of *Pmaip1*, *Bbc3*, and *Cdkn1a* were assessed as controls, and the expression of each gene was also examined in *Trp53* knockout lymphoma cells. Each treatment was performed three times, and the qRT-PCR analysis was undertaken with 3 technical replicates. Error bars represent standard error of the mean. Statistical tests were one-way ANOVAs with Šídák's multiple comparisons tests, performed to compare the indicated samples. Note that due to variation between biological replicates, some differences are not statistically significant despite obvious trends.

**Supplementary Figure 4. Further validation of loss of *Arrdc3* as providing a competitive advantage after TRP53 activation in the AH15A, AF47A, and 560 *Eμ-Myc* lymphoma cell lines.** (A) Cell cycle assay using the AH15A and 560 *Eμ-Myc* lymphoma cell lines with CRISPR/Cas9-mediated knockout of either *Arrdc3* or with NTsgRNA control lymphoma cells, treated with 5 μM nutlin-3a for 6 h. Each treatment was performed 3 times. Data are presented as means +/- standard deviation. (B) Example FACS plots from one replicate of AF47A cells treated with nutlin-3a from Figure 2A. Cells were gated to remove doublets, before comparing PI and Annexin V staining to assess viability. (C) Cell death assays using the AH15A and 560 *Eμ-Myc* lymphoma cell lines edited using CRISPR/Cas9 to ablate *Arrdc3*, treated for 24 h with the apoptosis-inducing drugs nutlin-3a, etoposide, or thapsigargin. Each lymphoma cell line treatment was performed 3 times, with 2 technical replicates each time. Data are presented as means +/- standard deviation. (D) Cell death assays using the AF47A *Eμ-Myc* lymphoma cell line edited using CRISPR/Cas9 to ablate *Bbc3* (encoding PUMA), treated for 24 h with the apoptosis-inducing drugs nutlin-3a or etoposide. Each treatment was performed 3 times, with 2 technical replicates each time. Data are presented as means +/- standard deviation. (E) Cell competition assays using the AH15A and AF47A *Eμ-Myc* lymphoma cell lines with CRISPR/Cas9-mediated knockout of either *Arrdc3* or *Trp53*, or with NTsgRNA-expressing or non-transduced Cas9-expressing lymphoma cell lines as controls. Cells were mixed (1:1) and treated with sub-optimal doses of either nutlin-3a (1.5 μM) or thapsigargin (1 nM) over a period of 14 days. Each competition assay was performed twice for lymphoma each cell line, with 2 technical replicates each time, with a representative example being shown. Data are presented as means +/- standard deviation.

**Supplementary Figure 5. Newborn *Arrdc3^-/-^* pups exhibit heart defects with incomplete penetrance.** (A-A’’’’’) Histological sections of a heart from a newborn *Arrdc3^+/+^* (i.e. wt) pup. (B-B’’’’’) Histological sections of a heart from a newborn *Arrdc3^+/-^* pup. (C-C’’’’’) Histological sections of a heart from one of two newborn *Arrdc3^-/-^* pups found to have a heart defect. Note the near total absence of the ventricle on the left in the images. (D-D’’’’’’) Histological sections from the heart of the second of two newborn *Arrdc3^-/-^* pups found with a heart defect. The images in D’-D’’’’’’ are close-ups of the approximate area outlined in D. Note the very small ventricular septal defect (arrows). Histological samples were stained with haematoxylin and eosin. Images were taken at the same magnification in A-C. Each image demonstrates a different section of the heart in frontal orientation.

**Supplementary Figure 6.** **The absence of** ***Arrdc3* has only minor impact on the behaviour of primary murine embryonic fibroblasts.** (A) qRT-PCR analysis performed to confirm the absence of *Arrdc3* expression in *Arrdc3^-/-^* MEFs, using *Gapdh* and *Atp5f1* as housekeeping genes. The experiment was performed once, and the qRT-PCR was undertaken with 3 technical replicates. (B) qRT-PCR to assess expression of *Arrdc3*, *Cdkn1a*, *Bbc3*, and *Pmaip1* in *Arrdc3^+/+^* MEFs that had been treated for 48 h with nutlin-3a (10 μM) or etoposide (2 μg/mL). *Gapdh* and *Atp5f1* were used as housekeeping genes. Each treatment was performed on 2 biological replicates, and the qRT-PCR was undertaken with 3 technical replicates. (C) Cell cycle analysis using MEFs derived from *Arrdc3^+/+^* and *Arrdc3^-/-^* E14.5 foetuses treated for 48 h with nutlin-3a (10 μM). Each treatment was performed 3 times, with 3 technical replicates for each (all plotted, data are presented as mean +/- standard deviation). (D) Cell death assay using MEFs derived from *Arrdc3^+/+^* and *Arrdc3^-/-^* E14.5 foetuses treated for 48 h with either nutlin-3a (10 or 20 μM) or etoposide (2 or 5 μg/mL). Each treatment was performed at least 3 times, with 3 technical replicates for each (all plotted, data are presented as mean +/- standard deviation).

**Supplementary Figure 7. The absence of *Arrdc3* has no apparent impact on haematopoietic cell development and viability.** (A-C) Flow cytometry analyses of haematopoietic cell development in the bone marrow, spleens, and thymi of lethally irradiated mice that had been transplanted with *Arrdc3^+/+^* (i.e. wt) or *Arrdc3^-/-^* E14.5 foetal liver cells. (A) Analysis of B cells in the bone marrow. At left, example FACS plot identifying the different B cell populations as determined by B220/IgM expression. At right, quantifications of cell numbers at the different stages of differentiation – pro-B/pre-B (B220^lo^, IgM^-^), immature B (B220^lo^, IgM^mid^), transitional B (B220^lo-hi^, IgM^hi^), and mature B (B220^hi^, IgM^mid^). No clear difference was observed between the two genotypes for any of the cell populations examined. (B) Analysis of B cells in spleens. At left, example FACS plot identifying the different B cell populations as determined by IgM/IgD expression. At right, quantifications of cell numbers at these different stages of differentiation – follicular B (IgM^+^, IgD^+^) and marginal B (IgM^lo^, IgD^+^). No clear difference was observed between the two genotypes for any of the cell populations examined. (C) Analysis of T cells in thymi. At left, example FACS plot identifying the different T cell populations as determined by CD4/CD8 expression. At right, quantifications of cell numbers at these different stages of differentiation – double-negative (CD4^-^, CD8^-^), mature (CD4^-^, CD8^+^ or CD4^+^, CD8^-^), and immature (CD4^+^, CD8^+^). No clear difference was observed between the two genotypes for any of the cell populations examined. (D) Viability assays of B cell populations purified from the bone marrow and spleen, or T cell populations from the thymus when treated with nutlin-3a (5 μM or 10 μM). No differences in viability were observed between the two genotypes. (E) Gene expression data from qRT-PCR analysis of splenic B cells and thymic T cells from lethally irradiated mice that had been transplanted with *Arrdc3^+/+^* (i.e. wt) or *Arrdc3^-/-^* E14.5 foetal liver cells, taken either at 0 h, or after 24 h of treatment with DMSO (vehicle control) or nutlin-3a (10 μM). Expression levels are normalised to *Gapdh* expression, and then to gene expression in one of the three *Arrdc3^+/+^* 0 h samples. *Arrdc3* expression was increased in the nutlin-3a treated *Arrdc3^+/+^* samples, and absent in the *Arrdc3^-/-^* samples, in both the B and T cells. Expression of *Cdkn1a* (*p21*) was used as a positive control for a TRP53 induced target gene, and it was strongly upregulated after treatment with nutlin-3a, as expected.

**Supplementary Figure 8. Immuno-phenotyping of the *Eμ-Myc^T/+^;Arrdc3^-/-^* lymphomas.**

(A) Blood cell content of lethally irradiated mice that had been transplanted with *Eμ-Myc^T/+^;Arrdc3^+/+^* or *Eμ-Myc^T/+^;Arrdc3^-/-^* E14.5 foetal liver cells, taken at time of sacrifice due to lymphoma burden. No significant differences were observed in the numbers of white blood cells (Student’s t-test, p>0.05 (p=0.7961)), red blood cells (Student’s t-test, p>0.05 (p=0.1622)), or platelets (Student’s t-test, p>0.05 (p=0.1307)) between the two genotypes. (B) Organ weights of the mice described in A. No significant differences were observed in the weights of the lymph nodes (Mann-Whitney test, p>0.05 (p=0.8968)), spleens (Student’s t-test, p>0.05 (p=0.6385)), or thymi (Mann-Whitney test, p>0.05 (p=0.8286)) between the two genotypes of reconstituted mice. (C) Immunophenotyping of lymphomas in the spleens of the mice described in A. Lymphoma cells were gated for single cells, live cells, B cells (B220^+^, CD19^+^), and then B cell stage (IgD and IgM). There is a clearly increased number of lymphomas predominantly consisting of IgD^-^, IgM^-^ cells in the recipient mice that had been transplanted with *Eμ-Myc^T/+^;Arrdc3^-/-^* E14.5 foetal liver cells compared to the lymphomas from the recipient mice that had been transplanted with *Eμ-Myc^T/+^;Arrdc3^+/+^* E14.5 foetal liver cells. Dotted lines represent average blood cell counts or organ weights of typical healthy C57BL/6 (wt) mice.
