# Supplementary material for: Genome-wide CRISPR screening identifies a role for ARRDC3 in TRP53-mediated responses
Source: Cell Death Differ. 2023 Dec 14;31(2):150–8. doi: 10.1038/s41418-023-01249-3 (PMC10850147; doi:10.1038/s41418-023-01249-3)
Supplement: Supplementary file 3 — Supplementary Methods, File Legends, Tables [file 41418_2023_1249_MOESM3_ESM.docx]

**SUPPLEMENTARY MATERIALS AND METHODS**

**Cell lines, isolation, and culture**

AH15A, AF47A, and 560 *Eμ-Myc* lymphoma cells were maintained in “FMA” medium (500 mL high-glucose Dulbecco’s modified Eagle’s medium (DMEM), supplemented with 10% (50 mL) foetal bovine serum (FBS, Sigma-Aldrich #F9423), 50 μM 2-mercaptoethanol (Sigma-Aldrich #M3148) and 100 mM asparagine (Sigma-Aldrich #A4284)). Cells were maintained at 37°C with 10% CO_2_ and passaged three times a week.

To obtain MEFs, E13 embryos were acquired, washed in 1x PBS (Gibco #14190-144), the head, foetal liver, and heart (where possible) removed, and the remaining tissue incubated in (at least) 1x trypsin (Lonza #BE02-007E) in PBS for ~30-60 min at 37°C with regular agitation. Cells were then strained, resuspended in media (500 mL DMEM with 10% FBS (Sigma-Aldrich #F9423), 5 mL GlutaMAX (Thermo Fisher Scientific #35050061), and 100 U/mL penicillin and 100 μg/mL streptomycin (Gibco #15140122)), washed with the same medium, and finally seeded onto previously prepared 10 cm dishes coated with 0.1% gelatin solution. Cells were maintained at 37°C with 10% CO_2_ and 3% O_2_ and passaged roughly twice a week.

All cell lines were routinely determined to be negative for *Mycoplasma* infection using a MycoALert detection kit (Lonza #LT07-118).

**Generating CRISPR/Cas9-capable *Eμ-Myc* lymphoma cell lines for screening**

AF47A *Eμ-Myc* lymphoma cells were first transfected with *Cas9* (*FuCas9Cherry*; Addgene #70182) then with the “Yusa” sgRNA library^1^. *Cas9* transfection was performed by generating HEK293T cells (ATCC #CRL-3216) transfected with FuCas9Cherry (10 µg) and lentiviral packaging constructs pMDL (5 μg), pRSV-REV (2.5 μg), and pVSV-G (3 μg) by calcium-phosphate precipitation as described previously^2^. *Eμ-Myc* lymphoma cells were transduced with viral supernatant containing 8 μg/mL polybrene (made in house) via spin infection (32°C, 2200 rpm, 2 h). Stably transduced cells (mCherry-positive) were sorted using an Influx flow cytometer (BD Biosciences).

YUSA library transfection was undertaken as above. Four independently infected replicates of AF47A-Cas9-Yusa cells were rapidly expanded to avoid premature selection of sgRNAs. sgRNA transfection efficiency was found to be 38-80% using an LSR IIW flow cytometer (BD Biosciences).

**CRISPR/Cas9 modifications of *Eμ-Myc* lymphoma** **cell lines**

Three *Eµ-Myc* lymphoma cell lines (AH15A, AF47A and 560) were transduced with Cas9, followed by an sgRNA expression vector (LV04; Sigma-Aldrich #CRISPR18V) targeting *Arrdc3* (C07: 5’-TCAGTTTGCCCCTCGTCATCGG-3’), as described above. Successful generation of indels in the targeted *Arrdc3* exon 6 were confirmed by next generation sequencing. A gene-specific PCR was performed using the primers (FWD 5’- GTTGTACGTGGAGTGCTGAC-3’ and REV 5’- TGTACCTTTCCTTAGCACATGTGA-3’) with the addition of overhangs to enable a second Illumina indexing PCR, as described previously^2^. The frequency of indels was calculated using the CRISPR indel calculator tool (<https://sarah-d.shinyapps.io/crispr-indel/>) (Fig. S2A). Three independent *Arrdc3*-targeting sgRNAs were initially tested via this method in the 560 *Eµ-Myc* lymphoma cell line, before selecting the sgRNA that gave the best indel results to generate the AH15A and AF47A cell lines as well. The AF47A *Bbc3*/PUMA knockout cell line (sgRNA sequence: 5’-CCAGCCCAGCAGCACTTAGAGT-3’) was prepared and validated in the same manner (Fig. S2B). Control cell lines were generated via transduction of a non-targeting sgRNA expression vector (FgH1tUTG_huBim_Ex3 (Addgene #85532)), and similarly validated.

**Cell death assays**

For MEFs, cells were seeded onto 12 well plates at 20,000 cells, in triplicate. The next day, cells were treated with either DMSO, nutlin-3a (10/20 μM, MedChemExpress #HY-10029), or etoposide (2/5 μg/mL, Ebewe Interpharma) for 48 h. For *Eμ-Myc* lymphoma cells, cells were seeded onto 96 well plates at 30,000 cells per well, in triplicate, then treated with either DMSO, nutlin-3a (1/2.5/5/10 μM), etoposide (2/10/20/50 ng/mL), or thapsigargin (0.0005/0.001/0.005/0.01/0.1/0.5/2 nM) (Sigma Aldrich #T9033) for 24 h. For bone marrow and spleen derived B cells, to remove unwanted cells, a biotinylated antibody cocktail was prepared containing GK1.5-biotin (CD4, 1:400 made in house), 53-6-7-biotin (CD8a, 1:400 made in house), S7-biotin (CD43, 1:400, BD Pharmagen #553269), and Ly76-biotin (Ter199, 1:400, made in house) in FACS buffer (1% FBS, 1% 0.5 M EDTA in PBS). After cells were removed from the single cell preparations of the tissues for intracellular FACS (see below), a small volume was set aside for a pre-sort purity check, and the remaining cells were centrifuged at 1500 rpm for 5 min, and resuspended in the antibody cocktail, and then incubated for 10+ mins at room temperature. The cells were then diluted up to 4 mL in FACS buffer, centrifuged at 1500 rpm for 5 mins, then resuspended in 100 μL FACS buffer. Then, 5 μL magnetic beads (Invitrogen #MSNB-6002-74) were added, and the samples incubated at room temperature for 5 min. Samples were then diluted up to 2 mL with FACS buffer, transferred into a FACS tube, and incubated in a magnet stand (STEMCELL Technologies #18000) for 5 min. The supernatant containing the cells of interest was then collected and transferred into a new tube, with a small volume again set aside for a post-sort purity check. Purity of the cell population was confirmed by staining cells for 30 min using antibodies against TCRβ (H57-597-PeCy7, BioLegend #109222) and CD19 (1D3-A700, made in house) diluted in FACS buffer, then washing in FACS buffer and assessing via FACS using an LSR IIW (BD Biosciences). Cells were then seeded onto 96 well plates at 50,000 cells per well, in triplicate, and treated with either DMSO or nutlin-3a (5/10 μM) for 0/6/24/48 h. Finally, cells were washed in PBS and resuspended in 1x Annexin V binding buffer (10x recipe: 0.1 M HEPES, pH 7.4, 1.4 M NaCl, 25 mM CaCl_2_, made in PBS) containing PI (1 μg/mL, Sigma-Aldrich #P4170) and Annexin V-A647 (1:1000-2000, made in house). Cells were analysed via FACS using an LSR IIW or Fortessa X-20 (BD Biosciences), and data were analysed using FlowJo (BD Biosciences) and Prism 9 (GraphPad).

**Cell cycle assays**

Cells were seeded in 6 well (MEFs) or 12 well (*Eμ-Myc* lymphoma cells) plates at either 50,000 cells (MEFs) or 500,000 cells (*Eμ-Myc* lymphoma cells). Cells were then treated with DMSO or nutlin-3a (10 μM). For MEFs, treatment was applied the day following seeding. Cells were then incubated for 48 h (MEFs) or 6 h (*Eμ-Myc* lymphoma cells), before being resuspended in a Foxp3/Transcription Factor buffer kit (Invitrogen #00-5523-00). Cells were counted to obtain equal numbers between samples, then washed in PBS, and incubated in LIVE/DEAD Fixable Far Red Dead Cell Stain (1:500, Invitrogen #L34973) for 30 min on ice. Cells were washed again, then incubated in Foxp3/Transcription Factor fixation buffer for 30 min on ice, washed again, and then incubated in permeabilisation buffer for at least 48 h at 4°C. Finally, cells were resuspended in DAPI (1 μg/mL) in PBS and examined via FACS using an LSR IIW (BD Biosciences). Flow cytometry data were analysed using FlowJo (BD Biosciences) and Prism 9 (GraphPad). Assays were analysed statistically using 2-way ANOVAs with Šídák's multiple comparisons tests.

**Cell competition assays**

Cells marked with different fluorescent markers were seeded in 6 well plates at 100,000 cells per well, per genotype. Cells were treated at approximate IC_20_ concentrations with either DMSO, nutlin-3A (1.5 μM), or thapsigargin (1 nM) (Sigma Aldrich #T9033), and then passaged every 2-3 days, and fresh drug applied at each passage. Cell proportions were quantified via FACS using an LSR IIW (BD Biosciences) at day 0, and then intervals of 2-3 days for the next 14 days. Data were then analysed using FlowJo (BD Biosciences) and Prism 9 (GraphPad).

**qRT-PCR**

*Eμ-Myc* lymphoma cells were prepared by seeding into 12 well plates at 1,000,000 cells per well and treated with DMSO, nutlin-3a (10 μM), or etoposide (40 ng/mL) for 6/24 hrs. MEFs were prepared by seeding into 6 well plates at 50,000 cells per well and treated (the next day) with DMSO, nutlin-3a (10 μM), or etoposide (2 μg/mL) for 48 hrs. All cells were additionally treated with the pan-caspase inhibitor Q-VD-OPh (25 μM). RNA was extracted from cell/tissues frozen in TRIzol (Thermo Fisher Scientific #15596018) as per manufacturer’s instructions. cDNA was then synthesised using SuperScript III First Strand Synthesis System (Thermo Fisher Scientific #11904018) as per manufacturer’s instructions. qRT-PCR assays were performed according to the manufacturer’s instructions using TaqMan Fast Advanced Master Mix (Thermo Fisher Scientific #4444557) and TaqMan Assay reagents targeting *Arrdc3* (#Mm00626887_m1), *Cdkn1a*/*p21* (#Mm00432448_m1), *Pmaip*/*Noxa* (#Mm00451763_m1), *Bbc3*/*Puma* (#Mm00519268_m1), *Hmbs* (#Mm01143545_m1), *Gapdh* (#Mm99999915_g1), and *Atp5f1* (#Mm05814774_g1). qRT-PCRs were run on a QuantStudio 12K Flex Real-Time PCR System (Thermo Fisher Scientific). Data were analysed using Prism 9 (GraphPad).

**HSPC infections and haematopoietic reconstitution of lethally irradiated mice**

Male C57BL/6-LY5.1/J mice (6-8 weeks old) were lethally irradiated with two doses of 550 Rad (for reconstitution with *Eμ-Myc* transgene-containing foetal liver cells. Mice were randomly assigned to experimental groups, and then intravenously injected with up to 500,000 foetal liver cells in 200 μL that had been washed and filtered. Mice were administered neomycin (2 mg/mL, Sigma-Aldrich #N1876) *ad libitum* via their drinking water for up to 4 weeks post-transplantation. To enable statistical comparison, >10 mice were transplanted with foetal liver cells from each genotype being compared.

**Intracellular FACS and immunophenotyping**

For splenic lymphoma samples from mice reconstituted with *Eμ-Myc^T/+^;Arrdc3^+/+^* or *Eμ-Myc^T/+^;Arrdc3^-/-^* foetal liver cells, tissues were thawed into FACS buffer, then centrifuged and resuspended again in FACS buffer, before being incubated in 50 μL antibody cocktail at 4°C with 10% 24G2 hybridoma supernatant containing rat monoclonal antibodies against Fcγ receptor (Fcγ block to prevent non-specific binding of antibodies to cells, made in house) (antibodies listed in Table S3), protected from light, for 20-60 min. Once stained, the samples were washed twice with FACS buffer, before being resuspended in 100 μL FACS buffer with PI (0.5 μg/mL). Samples were then analysed using an LSR IIW (BD Biosciences), FlowJo (BD Biosciences), and Prism 9 (GraphPad).

For experiments to assess the haematopoietic cell populations in irradiated mice that had been reconstituted with *Arrdc3^+/+^* (i.e. wt) or *Arrdc3^-/-^* foetal liver cells; bone marrow, spleen, and thymus were harvested ~10 weeks post-transplantation, homogenised, and washed with FACS buffer. Homogenates were made up to 10 mL with FACS buffer, and the cells counted using a TC20 Automated Cell Counter (Bio-Rad). 50-100 μL of homogenate was transferred to a 96 well round-bottom plate, centrifuged, washed with FACS buffer, then resuspended in 100 μL red fixable viability dye (1:500 in PBS, Cytek Biosciences #R760008), and incubated at room temperature, protected from light, for 20 min. Samples were then again washed in FACS buffer, then incubated at 4°C in 50 μL antibody cocktail with 10% Fcγ block, protected from light, for 30 to 60 min (antibodies listed in Table S4). Once stained, the samples were washed twice with FACS buffer, before being resuspended in 200 μL FACS buffer. Samples were the analysed using an Aurora (Cytek). Data were analysed using FlowJo (BD Biosciences) and Prism 9 (GraphPad).

Single-stained and negative controls were prepared for intracellular FACS using both cells and beads. Cells were prepared as above. Beads were prepared by incubating 1 drop of anti-rat IgG antibody coated beads (made in house) with the necessary antibody at 4°C, protected from light, for at least 15 min. Samples were then washed with and finally resuspended in 100-150 μL FACS buffer.

**Animal husbandry, tumour identification, and sacrificial processes**

Male *Eµ-Myc^T/+^;Arrdc3^+/-^* mice were crossed with female *Arrdc3^+/-^*_­_ mice and offspring were genotyped for *Eµ-Myc* transgene presence and *Arrdc3* zygosity. Mouse gender was also assessed via PCR where necessary. All primer sequences used for genotyping are presented in Table S2. Mice containing the *Eµ-Myc* transgene were monitored for lymphoma (enlarged spleen/lymph nodes, difficulty breathing, hunched, hind limb paralysis, weight loss) and sick mice were sacrificed at the predetermined humane endpoint as assessed by an experienced animal technician who was blinded to the genotype of the mice. Breeding mice were excluded from analyses. At the point of sacrifice, a retro-orbital bleed was taken, and blood cell counts were measured by Advia (Siemens). Mice were euthanised and enlarged, tumour-bearing organs (lymph nodes, spleen, and thymus) were collected, weighed, and frozen as single cell suspensions generated by mashing tissues through a 100 µM strainer. Lymphoma-free mouse survival curves, blood content, and organ weight data were statistically analysed using Prism (GraphPad).

**Histology and heart imaging**

Heart tissues from E19.5 pups were collected into 10% buffered formalin, paraffin embedded, serially sectioned in a frontal orientation, mounted on slides, de-paraffinised and stained with haematoxylin and eosin (H&E) by the WEHI Histology Lab. Tissue sections were imaged using a Stemi 2000-C microscope with an AxioCam HRc and AxioVision software (Zeiss).

**SUPPLEMENTARY FILE LEGENDS**

**Supplementary File 1. Phenotyping of E19.5 pups.** All information pertaining to the phenotyping of the *Arrdc3^+/+^*, *Arrdc3^+/-^*, and *Arrdc3^-/-^* pups at E19.5.

**Supplementary File 2. MAGeCK outputs from screen analyses.** Raw outputs from the different screen analyses that are shown in Figures 1A, S1A, and S1B. As shown in the figures, the data is sorted by “pos|rank”, which ranks the sgRNAs in terms of positive selection, corresponding to their p-values.

**SUPPLEMENTARY TABLES**

**Supplementary Table 1. Statistical comparisons for cell cycle assays.** Cell cycle assays were analysed statistically using 2-way ANOVAs with Šídák's multiple comparisons tests. Only the p-values and significance results for relevant comparisons are shown – for control and *Arrdc3^KO^* lymphoma cell lines between DMSO and nutlin-3a (5 μM) treatments, and for DMSO and nutlin-3a (5 μM) treatments between the control and *Arrdc3^KO^* lymphoma cell lines. *=p<0.05, **=p<0.01, ***=p<0.001.

|  | **DMSO *vs* nutlin-3a (5 μM)** | | | | | |
| --- | --- | --- | --- | --- | --- | --- |
| **Cell line** | **sgRNA target** | **sub-G_1_** | **G_1_** | **S** | **G_2_** | **post-G_2_** |
| **15A** | Non-targeting | >0.9999, n.s. | 0.0317, * | 0.0010, ** | 0.8049, n.s. | >0.9999, n.s. |
|  | *Arrdc3* | 0.9997, n.s. | 0.1464, n.s. | 0.1069, n.s. | 0.9994, n.s. | >0.9999, n.s. |
| **47A** | Non-targeting | 0.9999, n.s. | 0.0893, n.s. | 0.7737, n.s. | 0.0029, ** | >0.9999, n.s. |
|  | *Arrdc3* | 0.9984, n.s. | 0.0005, *** | 0.3273, n.s. | 0.0731, n.s. | 0.9997, n.s. |
| **560** | Non-targeting | >0.9999, n.s. | 0.0002, *** | 0.4523, n.s. | 0.0299, * | 0.9994, n.s. |
|  | *Arrdc3* | >0.9999, n.s. | 0.0035, ** | 0.0436, * | 0.9072, n.s. | >0.9999, n.s. |
|  | | | | | | |
|  | **NTsgRNA *vs* *Arrdc3.C07* sgRNA** | | | | | |
| **Cell line** | **sgRNA target** | **sub-G_1_** | **G_1_** | **S** | **G_2_** | **post-G_2_** |
| **15A** | DMSO | 0.9998, n.s. | 0.4019, n.s. | 0.5982, n.s. | 0.9959, n.s. | >0.9999, n.s. |
|  | Nutlin-3a (5 μM) | >0.9999, n.s. | 0.83138, n.s. | >0.9999, n.s. | 0.7066, n.s. | >0.9999, n.s. |
| **47A** | DMSO | >0.9999, n.s. | 0.9914, n.s. | 0.9988, n.s. | >0.9999, n.s. | >0.9999, n.s. |
|  | Nutlin-3a (5 μM) | 0.9999, n.s. | 0.7630, n.s. | 0.0547, n.s. | 0.8280, n.s. | >0.9999, n.s. |
| **560** | DMSO | >0.9999, n.s. | 0.0174, * | 0.0563, n.s. | >0.9999, n.s. | >0.9999, n.s. |
|  | Nutlin-3a (5 μM) | >0.9999, n.s. | 0.0117, * | 0.8298, n.s. | 0.3535, n.s. | >0.9999, n.s. |

**Supplementary Table 2. Primers used for mouse genotyping.** Primers used to assess mouse and embryo genotypes and genders via PCR.

| **Target** | **Forward (5’-3’)** | **Reverse (5’-3’)** | **Expected Band Sizes (bp)** |
| --- | --- | --- | --- |
| *Eµ-Myc* transgene | CAGCTGGCGTAATAGCGAAGAG | CTGTGACTGGTGAGTACTCAACC | ~900 |
| *Arrdc3* (wildtype) | AGCCAAGACTGGACTTGAATC | TTCACACCAAGGCTCTTTCC | 359 |
| *Arrdc3* (knockout) | ACTAACCGGATGCGTGAAAC | AAAAATTTCCCCATGAAAGC | 502 |
| Gender assessment | GATGATTTGAGTGGAAATGTGAGGTA | CTTATGTTTATAGGCATGCACCATGTA | XX: 685  XY: 280 |

**Supplementary Table 3. Antibodies used in lymphoma immunophenotyping.** List of antibodies used when immunophenotyping splenic lymphoma cells from *Eμ-Myc^T/+^;Arrdc3^+/+^* and *Eμ-Myc^T/+^;Arrdc3^-/-^* mice.

| **Clone** | **Target** | **Fluorophore** | **Dilution** | **Source** |
| --- | --- | --- | --- | --- |
| 5-1 | IgM | FITC | 1:400 | in house |
| 1D3 | CD19 | A700 | 1:400 | in house |
| 11-26c.2a | IgD | BV510 | 1:400 | BD Biosciences #563110 |
| RA3-6B2 | B220 | BV605 | 1:200 | BioLegend #103244 |

**Supplementary Table 4. Surface marker antibodies used in FACS.** List of antibodies used when conducting FACS assessments of the cellular makeup of bone marrow, spleens, and thymii from irradiated mice that had been reconstituted with an *Arrdc3^+/+^* (i.e. wt) or *Arrdc3^-/-^* haematopoietic system.

| **Clone** | **Target** | **Fluorophore** | **Dilution** | **Source** |
| --- | --- | --- | --- | --- |
| S450 | CD45.2 (Ly5.2) | A700 | 1:300 | in house |
| 5-1 | IgM | A647 | 1:400 | in house |
| 11-26c.2a | IgD | BV510 | 1:400 | BD Biosciences #563110 |
| 1D3 | CD19 | PE | 1:400 | in house |
| RA36B2 | B220 | FITC | 1:400 | in house |
| H57-597 | TcrB | PE.Cy7 | 1:400 | BioLegend #109222 |
| GK1.5 | CD4 | PercP-Cy5.5 | 1:800 | BioLegend #100434 |
| 53-6.7 | CD8 | BV650 | 1:400 | BioLegend #100741 |

**SUPPLEMENTARY REFERENCES**

1. Koike-Yusa H, Li Y, Tan EP, Velasco-Herrera Mdel C, Yusa K. Genome-wide recessive genetic screening in mammalian cells with a lentiviral CRISPR-guide RNA library. *Nat Biotechnol* 2014, **32**(3)**:** 267-273.

2. Aubrey BJ, Kelly GL, Kueh AJ, Brennan MS, O'Connor L, Milla L*, et al.* An inducible lentiviral guide RNA platform enables the identification of tumor-essential genes and tumor-promoting mutations in vivo. *Cell Rep* 2015, **10**(8)**:** 1422-1432.
